# Thrombin activity confinement and dense granule release drive the dynamics of arterial thrombus

**DOI:** 10.1371/journal.pcbi.1014062

**Published:** 2026-03-20

**Authors:** Efim S. Bershadsky, Dmitry Y. Nechipurenko

**Affiliations:** 1 Center for Theoretical Problems of Physico-chemical Pharmacology, Russian Academy of Sciences, Moscow, Russia; 2 Faculty of Physics, Lomonosov Moscow State University, Moscow, Russia; University of Southern California, UNITED STATES OF AMERICA

## Abstract

The mechanisms driving spatial heterogeneity of arterial thrombus and its three-stage dynamics are poorly understood. To investigate the potential principles regulating the size of the thrombus core and shell we developed a 3D continuum computational model that describes thrombus heterogeneity, thrombin-induced platelet dense granule secretion and clot propagation through thrombin and ADP-induced platelet activation. The continuum model predicted that spatial confinement of the thrombus core was a result of thrombin transport and a threshold-like dependence of platelet activation on thrombin concentration. This new model recapitulated three-stage dynamics observed *in vivo* and explained it with a burst-like ADP concentration dynamics due to the confinement of thrombus core propagation and rapid dense granule pool depletion within the core. The maximal shell size *in silico* was regulated by the transport of ADP and the kinetics of thrombin-dependent dense granules secretion. Simulations also predicted that partial propagation of thrombin inside the thrombus shell caused irreversible platelet activation by the low-dose thrombin and defined the residual shell size. Moreover, our results provided an explanation for the reduced size of a thrombus core observed in the mouse models of Hermansky-Pudlak syndrome. The continuum model was then applied to describe a FeCl_3_-induced thrombosis in macrocirculation, and described the thrombin-flux-depending switch between occlusive and non-occlusive thrombosis scenarios in mouse carotid artery. Finally, our simulations reinforced the hypothesis suggesting the importance of the large ADP-dependent thrombus shell for sealing the breach in case of a penetrating injury. Taken together, our results suggest a novel mechanism that may regulate arterial thrombus dynamics and offer several insights and сlarification to the core-and-shell model of arterial thrombus organization, as well as a possible role of the large thrombus shell in hemostasis.

## Author summary

Arterial thrombus is heterogeneous and exhibits complex spatiotemporal dynamics, however, the complete understanding of the underlying mechanisms is still missing. Using current knowledge of the key processes driving thrombus growth and the existing *in vitro* data on platelet responses, we developed a computational model that offered a new explanation of arterial thrombus dynamics observed *in vivo* through the combination of spatial confinement of thrombus core propagation and platelet dense granule pool depletion. Our results bring several new insights into the potential mechanisms that regulate thrombus structure and dynamics in both micro and macrocirculation and suggest an important role of thrombus shell in hemostatic response to the penetrating injuries.

## Introduction

Upon non-penetrating injury of arteries, venules and arterioles, either pathological occlusive or non-occlusive platelet-rich thrombus is rapidly formed. While occlusive thrombus is usually stable, non-occlusive thrombus normally demonstrates specific three-stage dynamics with rapid growth, followed by shrinkage and further stabilization [1,2]. Importantly, such dynamics and pronounced heterogeneity of thrombi were reported in both micro and macrocirculation of mice [2,3], though, the mechanisms underlying such a specific response are still poorly understood.

The heterogeneity of a thrombus formed upon laser-induced injury of mouse cremasteric arterioles is attributed to the spatial distribution of thrombin – a potent platelet activator that triggers multiple responses, including granule secretion [2]. The external thrombus shell is generally considered to form through platelet activation with weak agonists – like ADP and thromboxane A2, that are released in the core region of a thrombus possessing high thrombin activity. While the mechanisms responsible for the observed thrombus heterogeneity are more or less clear [2,4–9], the origin of a three-stage dynamics of the thrombus as well as the plateau-like dynamics (slow growth with saturation) of its core is not clear [10].

The complex dynamics of the external parts of an arterial thrombus depends on the interplay between hydrodynamic forces that tend to destabilize thrombus and opposing inter-platelet forces. The relatively weak level of platelet activation in the external parts of a thrombus was proposed to drive its fluid-like behavior due to the pronounced stochasticity of inter-platelet interactions in case when a low number of receptor-ligand complexes mediate these interactions [11]. Various mechanical aspects of thrombus stability against the flow have been recently studied [12,13], while the role of spatiotemporal dynamics of platelet agonists that modulate interplatelet interactions have gained less attention.

Several ideas have been proposed to explain the decrease of thrombus size, including the decrease of thrombin activity (for example, due to tissue factor inactivation or hindered substrate transport [14,15]), thrombus destabilization due to contraction-mediated redistribution of weakly interacting procoagulant platelets to the core-shell interface [16] and flow-mediated removal of the external part followed by clot stabilization due to low adhesivity of the fibrin-rich residual core [17].

To investigate the possible role of thrombin activity confinement and dense granule pool depletion in arterial thrombus dynamics that was recently suggested [18] using the particle-based model of thrombosis [19], we developed the new continuum model of thrombus formation. Based on the results obtained with this model, we suggest a new mechanism explaining the three-stage dynamics of a thrombus that does not postulate a decrease of thrombin activity on the considered timescales. Our simulations offer several new predictions, including a burst-like ADP concentration dynamics, the pronounced depletion of the dense granule pool in the core regions of a thrombus and importance of weak though irreversible activation of platelets by thrombin for stabilization of thrombus shell at the later stages of thrombogenesis.

## Results

### Three-stage dynamics of a thrombus shell *in silico* is the consequence of thrombus core confinement, granule pool depletion and the irreversible platelet activation by a low-dose thrombin

**Fig 1:**
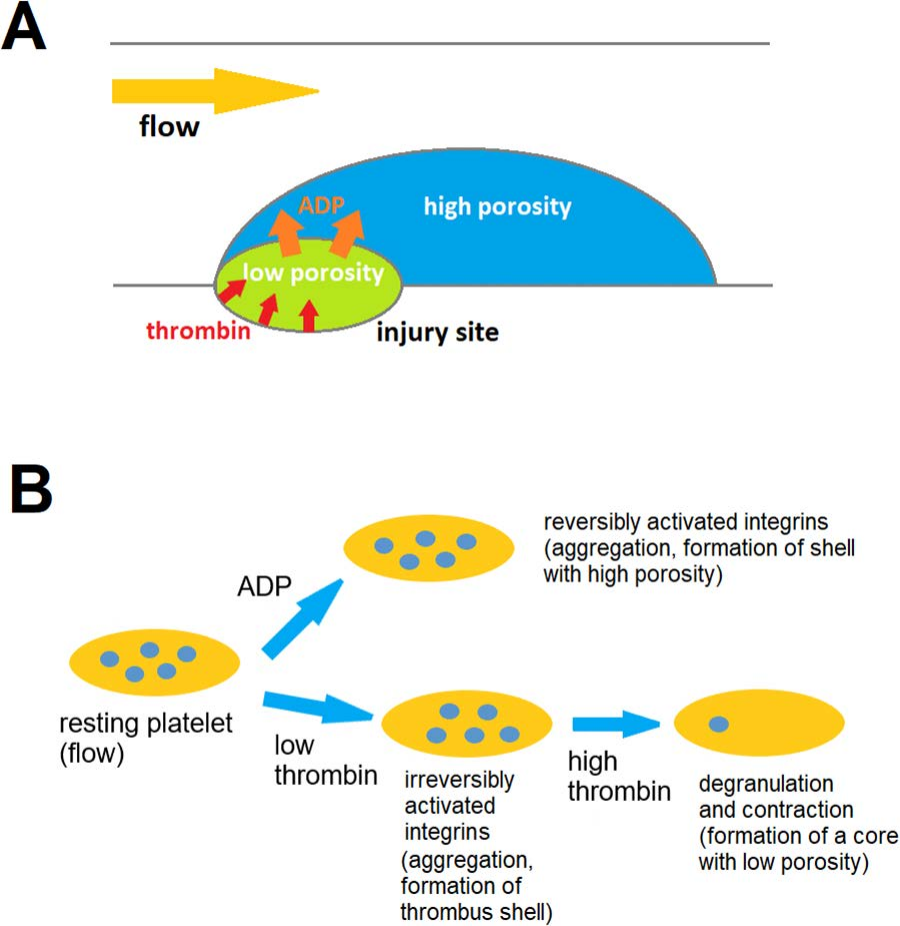
Illustration of the continuum model and its basic concepts. **A)** A continuum model considers a 3D vessel with an ellipsoidal injury site (with a zone of thrombin generation characterized by the time-dependent thrombin flux). Brinkman approach is used to calculate flow inside the thrombus and in the vessel. Thrombus shell and core zones are represented as porous domains with different porosities. Their location depends on the local concentrations of platelet activators- thrombin and ADP and degree of degranulation, as thrombin also triggers platelet dense granule secretion, which results in ADP release. Transport of thrombin and ADP is described by the convection-diffusion equation. **B)** In a continuum model the local platelets’ state depends on the local concentration of thrombin and ADP. ADP induces reversible platelet activation; low concentration of thrombin induces irreversible platelet activation; high concentration of thrombin additionally induces release of platelet dense granules with ADP, and transformation of a shell platelet into a core platelet if the particular degranulation threshold has been reached.

To get new insights into the specific dynamics of both thrombus core and shell observed *in vivo*, we built a novel 3D computational model that describes formation of a thrombus in a vessel using the continuum approach. The model takes into account irreversible platelet activation by thrombin, reversible platelet activation by ADP, platelet dense granule secretion in response to thrombin, heterogeneous structure of a thrombus consisting of the core and shell zone with different porosity values, as well as transport of thrombin and secreted ADP molecules (Fig 1, see also Methods section for details). The crucial model parameters were taken from the *in vitro* experiments on mouse platelets.

**Fig 2:**
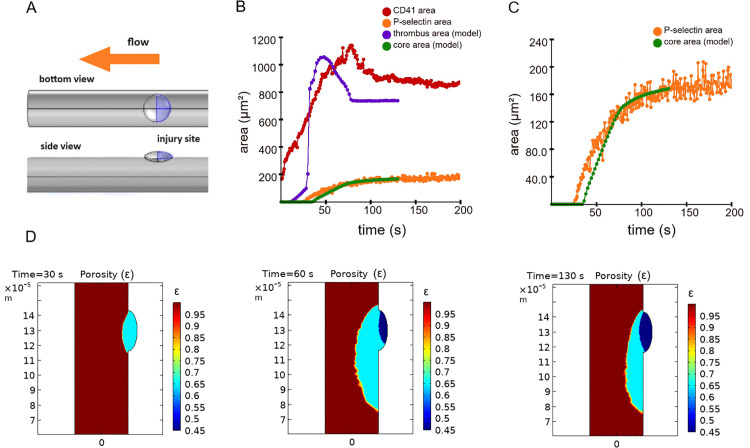
Comparison between the continuum model simulations and *in vivo* experiments on the laser-induced thrombosis in the wild type mice. **A)** Model geometry. 3D computational domain consisted of a cylinder representing the vessel and an injury site zone. Vessel diameter was 36 microns, while vessel length was 3060 microns. Injury zone was represented as an oblate ellipsoid with semiaxes of 13.5, 13.5 and 6 microns. Center of the ellipsoid was located on the vessel wall. Blue color marks the zone of thrombin generation. **B)** Temporal dynamics of thrombus core area and total thrombus area *in vivo* (data from Movie A [20], orange and red dots) and in the model simulation (green and blue dots). **C)** Temporal dynamics of the thrombus core area *in vivo* and in the model simulation (high resolution). **D)** Images of thrombus showing the core and a shell in the model simulation (vessel lumen is brown, shell is blue, core is dark blue). Flow direction was from top to the bottom. On each image color bar shows the values of porosity corresponding to the colors on this image. Note that thrombus core efficiently occupied the injury site zone.

**Fig 3:**
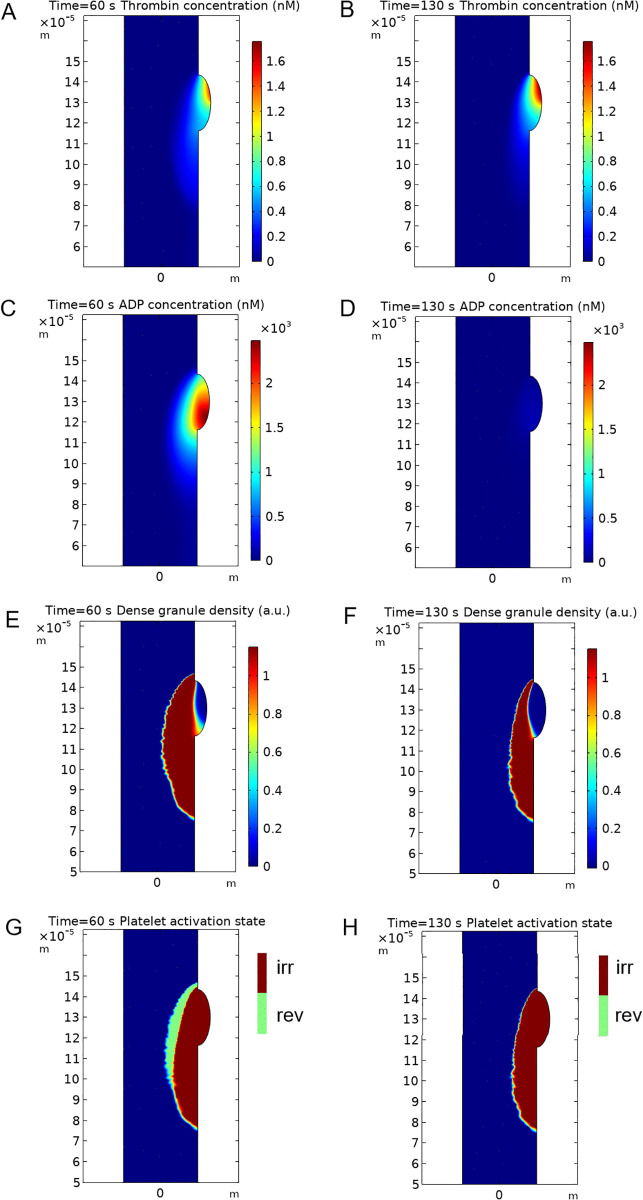
Continuum model profiles of thrombin and ADP, dense granule density and platelet activation state. On **A)-D)** color bar shows the values of concentration (units [nM]) corresponding to colors on this image. **A), B)-**Profiles of thrombin at 60 s and 130 s. **C), D) -** Profiles of ADP at 60 s and 130 s. Note the significant drop of ADP concentration in thrombus at 130 seconds. **E), F)-** Profiles of dense granule density inside the thrombus at 60 s and 130 s. Color bar shows the values of dense granule density (arbitrary units) corresponding to colors on the image. **G), H)-** Profiles of platelet activation state at 60 s and 130 s. Platelets reversibly activated by ADP are shown in green, platelets irreversibly activated by thrombin are shown in brown, vessel lumen is blue. Note the depletion of dense granules in a thrombus core at 130 s.

Using this new model, we simulated thrombus formation after the laser-induced injury of cremasteric mouse arterioles. Two external model parameters- equilibrium thrombin flux and characteristic time of thrombin generation (see Eq. 10) were inferred based on the comparison between model simulations and experimental video from [20](see details in Supplemental Text A in S1 Text). Localization of the thrombin generation zone in the upstream half of the injury zone was also chosen based on the comparison between model simulations with different possible locations of thrombin generation zone and the same experimental video from [20] (see Fig 2A, and details in Supplemental Text A in S1 Text). The calibrated model recapitulated both spatial and temporal dynamics of the thrombus core and shell observed *in vivo* (Fig 2 and S7, S1 and S2 Movies). Fast initial growth of the thrombus shell (Fig 2B) was the result of a burst-like secretion of dense granules with ADP from thrombin-activated platelets (Fig 3C and 3E).

The limitation of thrombus core propagation in the model (Fig 2B and 2D) was the consequence of two effects: 1) the presence of thrombin concentration gradient due to diffusion and convection-mediated transport of thrombin from the injury site (Fig 3A and 3B) and 2) the threshold-like response of platelets to thrombin that was recapitulated from the *in vitro* data [21] (S12 Fig). Importantly, these effects were responsible for several phenomena *in silico*: 1) the confinement of thrombus core growth in space 2) the pronounced localization of the region with depleted granule pool at the later stages of thrombogenesis (Fig 3F) – another prediction by the continuum model and, finally, 3) the decrease in ADP secretion rate, which was the consequence of the first two phenomena. As far as the core zone is by definition (see Methods section) the region where platelets actively secrete (or secreted) their granule contents, granule pool depletion and the confinement of a thrombus core resulted in a decrease of the total ADP secretion rate and hence – the drop in the overall ADP concentration (as ADP is a subject to both diffusion and convection) (Fig 3C and 3D). The decrease of ADP concentration due to both dense granule pool depletion and the confinement of thrombus core propagation resulted in a decrease of thrombus size, in line with the results obtained earlier with a particle-based model [19].

Formation of a large residual thrombus in the model (Fig 2D) occurred in a two-step process: 1) large initial thrombus shell formed due to ADP-induced platelet activation and 2) thrombin partly propagated inside this large thrombus shell and irreversibly activated platelets there (Figs 3G and S11). This part of a thrombus shell remained stable (Fig 3H) after the drop of ADP concentration in a thrombus (Fig 3D) but was passive in terms of granule secretion, because thrombin concentration was too low (Fig 3B and 3F).

Spatial localization of the thrombus core, which fully filled the injury site zone both in the model and experiment (Fig 2D) was the result of localization of thrombin generation zone in the upstream part of the injury site zone (Fig 2A and Supplemental Text A in S1 Text), and thrombin transport in a thrombus (Fig 3A and 3B).

### Thrombus core size and residual thrombus size are decreased in mouse models of Hermansky-Pudlak syndrome due to the lack of hydrodynamic protection of thrombin by the large initial thrombus

To better validate our model, we performed 3D simulations of thrombus formation following laser-induced injury in mice with Hermansky-Pudlak syndrome (also referred to as HPS mice).

The structure of the model and its parameters were the same as in the original model described above. The only change was to the initial value of releasable ADP in platelet N_ADP_, which was set to zero, because HPS mice lack normal secretion of dense granules containing ADP [20]. As a result, in the model simulations platelets did not release ADP. Model simulations were compared to the data from experimental video of laser-induced thrombosis in congenic B6.C3-Pde6brd1 Hps4le/J (light ear) mice (Movie D from [20]).

**Fig 4:**
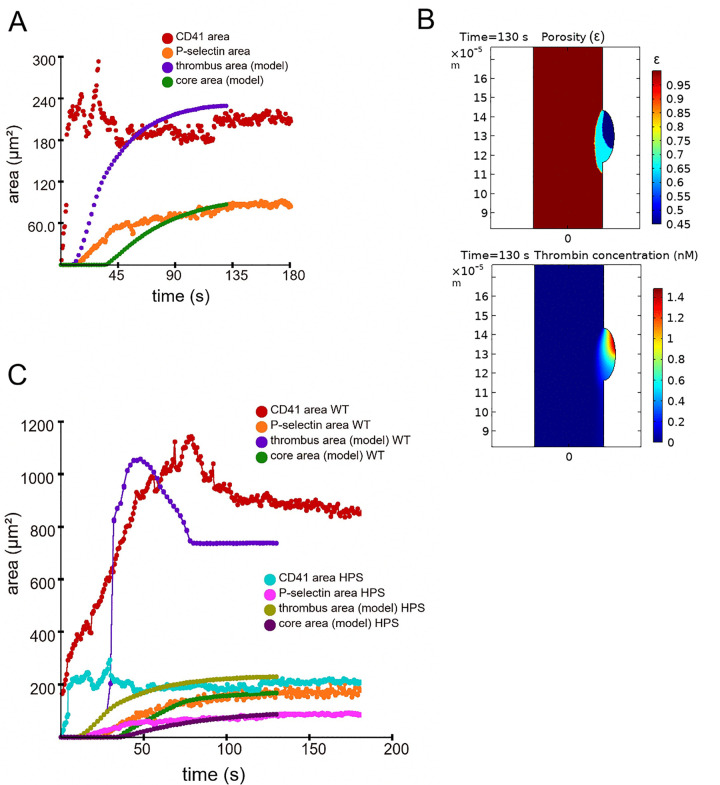
Comparison between the continuum model simulations and *in vivo* experiments on laser-induced thrombosis in mice with Hermansky-Pudlak syndrome. **A**) Temporal dynamics of thrombus core area and total thrombus area *in vivo* (light ear mouse, data from Movie D from [20], orange and red dots) and in the model simulation (green and blue dots). **B)** Upper image: image of a thrombus in the model simulation for HPS mouse at 130 s (vessel lumen is brown, shell is blue, core is dark blue). Flow direction was from top to the bottom. Color bar shows the values of porosity corresponding to colors on this image. Lower image: profile of thrombin in the model simulation for HPS mouse at 130 s. Color bar shows values of thrombin concentration (units [nM]) corresponding to colors on this image. Note the significant decrease of overall thrombus size and thrombus core size in HPS mouse compared to the wild type mouse. **C)** Overall data on temporal dynamics of thrombus core area and thrombus area *in vivo* (wild type mouse and mouse with Hermansky-Pudlak syndrome denoted as WT and HPS; data from Movie A (orange and red dots) and Movie D (light magenta and robin egg blue dots) from [20]; and in the model simulations (green and dark blue dots for WT mice; dark purple and dark olive dots for HPS mice).

The model calculations showed quantitative agreement with the experimental dynamics of thrombus formation, with the exception of the short initial phase of growth (Fig 4A and S3 and S4 Movies). The position of the zones of the thrombus core and the thrombus shell relative to the injury site were also similar in the experiment and the model (Fig 4B).

As expected, calculations showed that in the absence of ADP release, the formation of a large thrombus shell did not occur. The lack of hydrodynamic protection from the thrombus shell led to a change in the thrombin profile in the thrombus and a decrease in its maximum concentration in the thrombus (compare Figs 4B and 3B). As a result, both the thrombus core area and the residual thrombus area were significantly reduced compared to the calculation results for the wild-type mice (Fig 4C).

Taken together, our simulations predicted that both thrombus core size and residual thrombus size are decreased in case of an abrogated dense granule secretion due to the lack of a hydrodynamic protection of thrombin by the large initial thrombus.

### Thrombin flux from the injury site defines the scenario of thrombus growth in a FeCl_3_-induced thrombosis in a carotid artery

**Fig 5:**
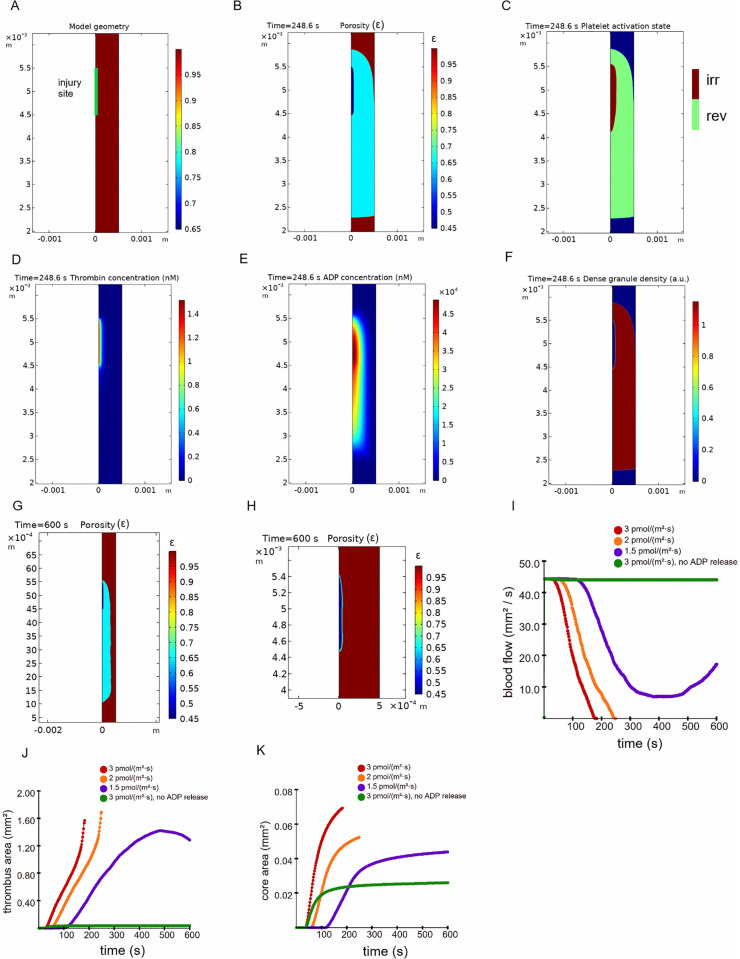
Mechanism of thrombus growth and occlusion in the *in silico* simulations of FeCl_3_-unduced thrombosis. **A)** Model geometry. 2D computational domain consisted of a rectangle representing the vessel and a segment representing the injury site zone. Vessel height was 0.5 mm, vessel length was 42.5 mm. Injury site length was 1 mm. Injury site zone coincided with the zone of thrombin generation. Green color marks the injury site zone. **B)-F)** show the data from the same simulation which ended in vessel occlusion at 248.6 second. Maximal thrombin flux was 2 pmol/(m^2^ ⋅ s). At all of the images flow direction was from top to the bottom. **B)** Image of the thrombus at the time of occlusion. Vessel lumen is brown, thrombus core is dark blue, while thrombus shell is blue. Color bar shows the values of porosity corresponding to colors on the image**. C)** Platelet activation state at the time of occlusion. Platelets reversibly activated by ADP are shown in green, platelets irreversibly activated by thrombin are shown in brown, vessel lumen is blue. **D)** The profile of thrombin at the time of occlusion. Color bar shows the values of concentration (unit [nM]) corresponding to colors on the image **E)** Profile of ADP at the time of occlusion. Color bar designations correspond to panel “**D**”. **F)** Profile of dense granule density inside the thrombus at the time of occlusion. Color bar shows the values of dense granule density (arbitrary units) corresponding to the colors on the image. **G)** Thrombus growth at intermediate value of maximal thrombin flux. Image of a non-occlusive thrombus at 600 seconds. Vessel lumen is brown, thrombus core is dark blue, while thrombus shell is blue. Maximal thrombin flux was 1.5 pmol/(m^2^ ⋅ s). Color bar designations correspond to panel “**B**”. **H)** The effect of turning off the ADP release from platelet dense granules on thrombus growth. Image of a thrombus at 600 seconds. Vessel lumen is brown, thrombus core is dark blue, while thrombus shell is blue. Maximal thrombin flux was 3 pmol/(m^2^ ⋅ s). Color bar designations correspond to panel “**B**”. **I)** Temporal dynamics of a blood flow in the vessel *in silico*. Red line: simulation with maximal thrombin flux of 3 pmol/(m^2^ ⋅ s). Orange line: simulation with maximal thrombin flux of 2 pmol/(m^2^ ⋅ s). Blue line: simulation with maximal thrombin flux of 1.5 pmol/(m^2^ ⋅ s). Green line: simulation with maximal thrombin flux of 3 pmol/(m^2^ ⋅ s), when the ADP release from platelet dense granules was turned off. **J)** Temporal dynamics of the thrombus area in the model simulations. Color designations correspond to panel “**I**”. **K)** Temporal dynamics of the thrombus core area in the model simulations. Color designations correspond to panel “**I**”.

To investigate the regimes of thrombus formation in the large vessels, we performed simulations of the FeCl_3_-induced thrombosis in mouse carotid artery using a 2D version of the continuum model (Fig 5; see details in Methods section, ‘2D model of thrombogenesis’). In experiments, the severity of the injury and the dynamics of thrombus formation strongly depend on the concentration of the applied FeCl_3_ [22,23]. To reproduce the effects of different degrees of the injury, we performed simulations with different values of the thrombin flux.

At high values of thrombin flux (2–3 pmol/(m^2^ ⋅ s)), the model predicted vessel occlusion (Fig 5B and 5I and S5 and S6 Movies). Expectedly, time to occlusion decreased with increasing thrombin flux (Table 1). The model predicted that thrombus penetration into the vessel and subsequent occlusion were the result of platelet activation in response to ADP (Fig 5C). The high concentration of ADP in the thrombus was due to the secretion of dense granules by thrombin-activated platelets (Fig 5E, 5D, and 5F).

**Table 1:** Scenarios of FeCl3-induced thrombosis in 2D model simulations. * Simulations were stopped at the moment of occlusion or, if no occlusion was observed during 600 seconds, simulation lasted 600 seconds.

Thrombin flux (pmol/(m^2^ ⋅ s))	Time to occlusion (s)	Maximal thrombin concentration (nM) (at the end of the simulation*)	Remarks
3	182.2	2.25	
2	248.6	1.52	
1.5	No occlusion	1.15	Large thrombus
1	No occlusion	0.091	No thrombus
3, ADP release turned off	No occlusion	1.71	Small thrombus near the surface of the injury site

Interestingly, model predicted expansion of thrombus core after the occlusion (S8 Fig and Supplemental Text B in S1 Text), however, these simulation results should be interpreted with caution.

At intermediate values of thrombin flux (1.5 pmol/(m^2^ ⋅ s)), a subocclusive thrombus regime was observed (S7 Movie and Fig 5G). The thrombus grew large, almost reaching the opposite wall of the vessel. However, after reaching the peak, the thrombus size began to decrease (Fig 5J). Eventually, vessel occlusion was not achieved, and the flow in the vessel was gradually restored (Fig 5I).

At low values of thrombin flux (1 pmol/(m^2^ ⋅ s)), thrombus growth was absent (S12 Fig). This was explained by the fact that the maximum concentration of thrombin did not reach the threshold of platelet activation in the model (see Table 1).

Turning off the secretion of ADP by platelets led to the formation of a small thrombus near the injury site zone (Fig 5H and 5J).

All these predictions of the model are in qualitative agreement with the experimental data [22–24].

### Large thrombus shell might be important for reaching the occlusion in response to the penetrating injury

Our simulations show that the transient build-up of a large thrombus shell is the result of the interplay between thrombin activity and spatiotemporal dynamics of dense granule secretion. However, the physiological role of such transient and massive thrombus shell is not clear.

**Fig 6:**
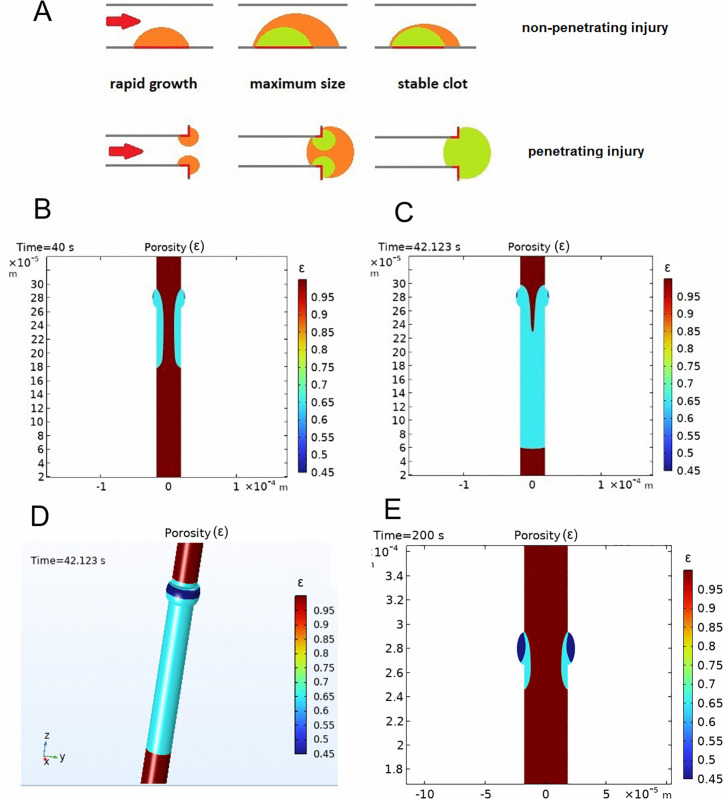
Simulation of thrombus growth after the circular vessel injury in 3D. **A)** Illustration of the idea suggesting the importance of the large thrombus shell for hemostasis in response to the penetrating injury. Top row: three stages of thrombus formation after the non-penetrating injury. Thrombus becomes smaller due to the decrease in ADP concentration. Thrombus shell is shown in orange, while the core is shown in green. Bottom row: three stages of the hemostatic plug formation upon complete vessel dissection: formation of a large shell allows the thrombus to reach itself from the opposite sides and thus completely occlude the injured vessel. **B)-E)** 2D axisymmetric computational domain consisted of a cylinder representing the vessel and the injury site zone. Vessel diameter was 36 microns, vessel length was 3060 microns. Injury zone was represented as a torus with an elliptic section with semiaxes of 13.5 and 6 microns. **B), C)**- Images of thrombus in the model simulation at 40 seconds and at the moment of occlusion (42.123 s). Vessel lumen is brown, shell is blue, while the core is dark blue. Flow direction was from top to the bottom. On each image color bar shows the values of porosity corresponding to colors on this image. **D)** 3D view of the thrombus at the moment of occlusion. Vessel lumen is brown, shell is blue, core is dark blue. **E)** Simulation where ADP release from platelet dense granules was turned off. Image of the thrombus in the model simulation at 200 seconds. Vessel lumen is brown, shell is blue, while the core is dark blue. Flow direction was from top to the bottom. Color bar shows values of porosity corresponding to colors on this image.

We hypothesized that a burst-like release of the secondary messengers (like ADP) and consequent build-up of a massive though transient structure allows the system to scan the environment and ultimately select between a non-occlusive response (preferred in case of a non-penetrating injury that can be associated with thrombosis) and occlusive response (optimal in case of a penetrating injury and associated with a hemostasis). If the surface of a growing platelet aggregate does not “touch” itself due to the geometry of the injury, it will eventually decrease in size due to the decrease of ADP concentration (Fig 6A, top). However, in case of a penetrating injury the hemostatic aggregate that grows in several directions can close on itself if it’s big enough, resulting in a vessel or injury closure (Fig 6A, bottom).

To illustrate this idea we performed simulation of thrombus formation in response to the circular injury of the vessel, which mimics the hemostasis scenario, i.e., when a thrombus can “connect” to itself (see details in Methods section, ‘2D axisymmetric model of thrombogenesis’). In this case thrombus became occlusive already at the 42nd second of the simulation as it rapidly closed the vessel growing towards itself (Fig 6B, 6C, and 6D). Importantly, in this simulation we used the same value of thrombin flux as in the simulations describing non-penetrating laser-induced thrombosis model (Fig 2), where thrombus was not occlusive and displayed a three-stage dynamics. In line with our idea, turning off granule secretion and hence ADP-dependent thrombus shell formation resulted in small circular thrombus that could not occlude the vessel (Fig 6E). These results reinforce the hypothesis suggesting that formation of a large thrombus shell is crucial in the settings of hemostasis.

## Discussion

In this work we used *in silico* approach to investigate the mechanisms governing a three-stage dynamics of thrombi formed after the non-penetrating injuries in both micro- and macrovessels *in vivo*. We suggest a new mechanism that explains the decrease of thrombus size by the decrease of the ADP concentration within the thrombus due to the dense granule pool depletion in the core regions of a thrombus and the confinement of thrombus core propagation. In our simulations a high thrombin activity within the inner part of a thrombus triggers rapid degranulation leading to the burst of ADP concentration and rapid platelet aggregation. As platelet activation with ADP is reversible in our models (reflecting the known phenomenon of platelets disaggregation *in vitro* in case of the decrease in ADP concentration [25]), the drop of ADP concentration due to both the confinement of thrombus core and the dense granule pool depletion within the core is followed by the partial disassembly of the thrombus. The continuum model offered a simple explanation of the plateau-like dynamics of the thrombus core propagation: combination of plateau-like kinetics of thrombin generation, thrombin concentration gradient and a threshold-like response curve of platelets to thrombin results in a saturation of thrombus core growth. It is actually this confinement of thrombin activity that leads to the limitation of thrombus core propagation and a consequent drop of ADP secretion rate that allowed as to explain a three-stage dynamics of a thrombus. As platelets activation with thrombin is considered irreversible in our models (reflecting the fact that thrombin irreversibly cleaves PAR receptors on the platelet surface [26]), our simulations predict that irreversible activation of platelets with low concentration of thrombin is responsible for the large size of the residual thrombus observed in the laser-induced injury model of mouse cremaster arterioles. Hence, our models also offered an important modification to the existing core-and-shell paradigm, explaining the large size of the shell region in the later stages of thrombus formation by the cumulative effects of low thrombin activity in this region, but not the action of weak platelet agonists (like ADP or TXA2). Our simulations also predict a distinct localization of the region with depleted granule pool that is a result of thrombin concentration gradient and the non-linear dependence of dense granule secretion kinetics on thrombin concentration. Interestingly, this prediction is qualitatively in line with the experimental data on spatial distribution of platelets that secreted alpha-granules *in vivo*, showing a distinct P-selectin-positive core region and minimal (if any) сore-to-shell transition zone [2].

Importantly, the suggested mechanism explaining a decrease in thrombus size by the combination of spatial distribution of thrombin, confinement of thrombus core propagation and the dense granule pool depletion does not imply the rapid decrease of a thrombin flux that was postulated in other works [27]. We believe that this result is important, since it is unlikely that thrombin activity decreases significantly at the timescales of 1–3 minutes, even if its production rate decreases, because thrombin specifically binds to both platelets [7,28] and fibrin [29]. Moreover, a large volume of the experimental and clinical data indicate the important role of secondary hemostasis reactions at the later stages of thrombus formation, associated with the stabilization of thrombi and hemostatic plugs. All these data, in our opinion, make the assumption of the rapid decrease in thrombin activity within a thrombus implausible.

### Model limitations

The current version of the model has several important limitations: it does not describe platelet transport to the thrombus (that may lead to the overestimated values of thrombus growth rate when ADP is released in a thrombus core), does no account for thrombus shape change due to the mechanical effects (both thrombus plasticity and disruptions). It also postulates certain dynamics of the thrombus flux from the injury site (see Eq. 10) and does not account for thrombus contraction that leads to increase in platelet packing density (a mechanism through which platelets in the outer layers can be dragged to the core region with high thrombin concentration and start to secrete their granule content). It also does not account for thrombin binding to platelets and fibrin within the thrombus and the hindered diffusion of molecules in a thrombus core [4] that might quantitatively affect thrombin distribution within the thrombus. Model also does not account for vessel elasticity.

Since hindered thrombin diffusion and burst-like dynamics of thrombin flux from the injury site were discussed in the literature as major possible regulators of thrombus dynamics [14,15,27], we performed several additional simulations which considered these effects (see Supplemental Text A in S1 Text). These simulations demonstrated quantitative, but not qualitative effect on the dynamics of thrombus formation comparing to the basic model.

Our model did not reproduce the initial 30-second period of thrombus growth in laser-induced injury. Importantly, similar period of growth is observed in the presence of thrombin inhibitor hirudin [2], which suggests that this process is controlled via thrombin-independent pathways. These pathways may include release of platelet agonists from injured endothelial cells or collagen-induced platelet activation, which were not considered in the model.

In general, we believe that all these limitations are not critical for achieving the main goal of this study: investigate the new potential mechanism that regulates thrombus dynamics.

### Predictions and conclusions

In our model the confinement of the core was the result of plateau-like kinetics of thrombin generation, localization of thrombin generation zone, thrombin transport within the thrombus, and a threshold-like response of platelets to thrombin concentration. In contrast, Stalker and colleagues suggested that limited propagation of thrombin inside the thrombus was the result of the action of the thrombin inhibitors [2]. We performed additional simulation and compared the dynamics of thrombus growth in the presence and in the absence of thrombin inactivation by plasma inhibitors. The results were almost identical (S4 Fig), because thrombin retention time within thrombus is much less than the characteristic time of thrombin inhibition. We conclude that thrombin inactivation by plasma inhibitors does not affect significantly the dynamics of arterial thrombus growth in this particular model. However, inactivation of factors Va and VIIIa by activated protein C was shown to be an important factor limiting thrombus formation in cremaster laser-induced injury model [30] that likely affects both the magnitude and kinetics of thrombin flux from the injury size and should be analyzed separately using a more detailed model.

Meng and colleagues suggested that decrease of the thrombus core size in mice with Hermansky-Pudlak syndrome reflects the decreased sensitivity of a granule secretion to thrombin that they observed *ex vivo* [20]. In contrast, in our model we did not suggest any change of platelet sensitivity to thrombin in mice with Hermansky-Pudlak syndrome compared to the wild type mice. Significant decrease of thrombus core size was due to the decrease of thrombin propagation inside thrombus in the absence of hydrodynamic protection by large thrombus shell. This idea also allowed to explain why thrombus core is significantly decreased in HPS mice, but not in mice treated with P2Y12 antagonist cangrelor (compare data from [20] and [31]). In mice treated with cangrelor, the maximal thrombus area was significantly larger than in HPS mice. As a result, sufficient level of hydrodynamic protection of thrombin was likely present, which possibly allowed thrombin to form a larger thrombus core.

In FeCl_3_-induced thrombosis of mouse carotid artery the scenario of thrombus growth and time to occlusion depend on the degree of injury induced by different concentration of the applied FeCl3 [22,23]. In mice with Hermansky-Pudlak syndrome, which lack normal platelet secretion of ADP, the thrombus does not grow at all [24]. In wild type mice, when platelet activation in response to ADP is inhibited by clopidogrel, the thrombus grows to a very small size [22]. Our computations of thrombus growth in FeCl_3_ – induced thrombosis have qualitatively reproduced all of these effects.

Our model predicts rather low peak thrombin concentration in a thrombus of about 2–3 nM. Cornelissen and colleagues demonstrated that mouse platelets are very sensitive to thrombin, and have low thrombin EC50 value of 0.1 nM, compared to about 1 nM for human platelets [32]. High concentrations of thrombin are usually observed in the absence of flow in the *in vitro* tests (like TGA) – however, under arterial shear rate conditions of 1000–2000 s^-1^ it is generally recognized that local thrombin concentrations can be in nanomolar range both *in vitro* and *in vivo*. This provides a possible explanation for the physiological relevance of such a high sensitivity of platelets to thrombin.

Our simulations reinforce the idea that large thrombus shell is important in the settings of hemostasis: during primary response to the penetrating injury the thrombus may grow towards itself due to the geometry of the injury. Formation of a large shell may allow thrombus to connect to itself like in our simulations (Fig 6), thus sealing the breach. In line with this hypothesis, dense granule pool deficiency results in bleeding complications in both humans [33] and the mouse models of HPS [24]. Another important argument is the failure to form hemostatic plug under the effect of P2Y12 antagonist upon the severe injury in a mouse jugular vein puncture model [34].

There are several model predictions that can be tested experimentally. First, our computations predict that thrombus core platelets are degranulated in their dense granule content at 2 minutes after laser-induced injury, while shell platelets preserve their dense granules. Second, our computations predict that both core and shell platelets are irreversibly activated by thrombin (i.e., their PAR receptors are cleaved) at 2 minutes after laser-induced injury. Finally, our computations estimate maximal thrombin concentration in thrombus at 1–3 nM both in laser-induced and FeCl_3_-induced injury.

Taken together, our results give several new insights into the potential mechanisms that drive the dynamics of arterial thrombus in both micro- and macrovasculature, offer a modification to the basic core-and-shell concept through emphasizing the possible role of the irreversible activation of platelets by the low-dose thrombin in a thrombus shell and also suggest that large ADP-dependent thrombus shell is crucial in the settings of hemostasis.

## Methods

### 3D continuum model of thrombogenesis

In this work we describe a novel 3D computational model for analyses of the spatiotemporal evolution of a thrombus in a mouse cremaster arteriole.

The model takes into account the following phenomena: heterogeneous structure of the thrombus consisting of a core and a shell; reversible activation of platelets in response to ADP; irreversible activation of platelets in response to thrombin; secretion of dense granules with ADP from thrombin-activated platelets. This new model allows us to take into account the relationship between thrombus formation and the distribution of soluble platelet activators within the thrombus.

The continuum model is based on a set of partial differential equations. The thrombus was considered as a porous medium consisting of 2 compartments with different porosity values corresponding to the core and shell of the thrombus. Flow in the vessel and inside the thrombus was described using Brinkman approach (see below). Thrombin and ADP were considered as the primary platelet agonists and their concentration dynamics were computed via convection-diffusion transport models. Agonist concentrations were used to determine the evolution of local platelet activation status and thrombus porosity as well as the dynamics of ADP release from platelets in a two-compartment model approach. The key activation process in the model is platelet dense granule secretion: the degree of platelet degranulation determines platelet activation status, which, in turn, impacts local porosity values (see below). Solving the system of model equations allows to infer the dynamics of thrombus formation as a function of both external and internal model parameters. All model equations were solved using the COMSOL Multiphysics software package [35]. The model predictions were compared with experimental data in the form of previously published videos of thrombosis in mouse arteriole models.

### General description

#### Computational fluid dynamics.

**Model geometry and hydrodynamics:** The model geometry reproduced the vessel with the injury site zone from a representative video of laser-induced thrombosis in microvasculature [20]. The computational domain consisted of a cylindrical vessel with a diameter of 36 microns and a length of 3060 microns and an injury site zone. Such vessel geometry has the diameter/length ratio of 0.0117, in line with the geometry described in [36] to account for the correct hemodynamic conditions with constant pressure drop boundary conditions. The injury site zone was described as the difference between an ellipsoid with semi-axes of 13.5, 6 and 13.5 microns and a cylindrical vessel (Fig 2A). The center of the ellipsoid was located on the wall of the vessel. The pressure drop was 1100 Pa to ensure a wall shear rate of 1000 s^-1^ in a vessel without thrombus.

Thrombus permeability: The thrombus was considered as a porous medium consisting of 2 compartments with different porosity values corresponding to the core and shell of the thrombus [2]. The hydraulic permeability of the core and shell zones were calculated based on their porosity values according to the Kozeny-Carman equation [37]. We used the following equation:


$$\kappa =\;\epsilon^{\text{3}}\cdot {\text{D}}_{\text{p}}^{\text{2}}/(\text{180}\cdot (\text{1}-\epsilon {)}^{\text{2}}),$$ (1)

where  κ is the permeability,  ϵ is the porosity, ${\text{D}}_{\text{p}}$ is the average platelet diameter (2 µm). The values of all model parameters are given in Table 2.

**Table 2:** Model parameters. Designations: MPV - mean platelet volume, WSR - wall shear rate.

Name	Symbol	Value	Comment/Reference
**Simulation**			
Total time of 3D simulation	T_3D_	130 s	
Total time of 2D simulation	T_2D_	600 s	
Total time of 2D axisymmetric simulation	T_2D_ax_	45-200 s	
Mesh update time	T_mesh_	2 s	
**Thrombus structure**			
Thrombus shell porosity	shPor	0.65	[2]
Thrombus core porosity	corePor	0.45	[2]
**Platelet activation by agonists**			
Threshold concentration of thrombin	v_1_	0.1 nM	[32]
Threshold concentration of ADP	v_2_	0.09 µM	[32]
**Formation of thrombus core**			
Initial value of variable w, corresponding to normalized platelet granule content	w_0_	1	
Threshold value for transformation of shell platelet into core platelet	v_3_	0.368	w_0_/e, where e ≈ 2.718, Euler’s number
**Diffusion coefficients of agonsists**			
Thrombin diffusion coefficient	D_IIa_	67 µm^2^/s	[38], corrected for blood plasma viscosity
ADP diffusion coefficient	D_ADP_	237 µm^2^/s	[39]
**Thrombin generation and inactivation**			
Characteristic time of thrombin generation	t_0_	30 s	Fitted parameter
Thrombin flux in laser-induced injury	J_max_	12 pmol/(m^2^ ⋅ s)	Fitted parameter
Thrombin flux in FeCl3-induced injury	J_max_	1-3 pmol/(m^2^ ⋅ s)	Varied parameter
Constant of thrombin inactivation by plasma inhibitors	k_i_	0.025 s^-1^	[40]
**Thrombin-induced ADP secretion**			
Lower threshold of thrombin concentration	c_1_	0.4 nM	[21]
Upper threshold of thrombin concentration	c_2_	0.8 nM	[21]
Maximal ADP secretion rate	K_max_	0.15 s^-1^	[41]
Total amount of releasable ADP in mouse platelet	ADP_0_	0.46 nmol/10^8^ platelets	[42]
Concentration of releasable ADP in mouse platelet	N_0_	1.15 mol/m^3^	ADP_0_/MPV
**Platelet**			
Mouse platelet volume	MPV	4fl	[43]
Mouse platelet diameter	D_p_	2 µm	Based on MPV
**Blood rheology**			
Blood plasma dynamic viscosity	η_1_	1.25 mPa ⋅ s	[44]
Whole blood dynamic viscosity	η_2_	3.4 mPa ⋅ s	[45]
**3D model geometry (arteriole)**			
Vessel diameter (arteriole)	D_art_	36 µm	Movie A from [20]
Vessel length	L_art_	3060 µm	Based on D_art_ and results from [36]
Injury site length	L_inj_	27 µm	Movie A from [20]
Injury site depth	D_inj_	6 µm	Movie A from [20]
**2D model geometry (carotid artery)**			
Vessel diameter	D_car_	0.5 mm	[23]
Vessel length	L_car_	42.5 mm	Based on D_car_ and results from [36]
Injury site length	L_inj2_	1 mm	[23]
**2D axisymmetric model geometry (arteriole)**			
Vessel radius	R_art_	18 µm	Movie A from [20]
Vessel length	L_art_	3060 µm	Based on R_art_ and results from [36]
Injury site length	L_inj_	27 µm	Movie A from [20]
Injury site depth	D_inj_	6 µm	Movie A from [20]
**Hydrodynamics**			
Wall shear rate	WSR	1000 s^-1^	[46]
Pressure drop in 3D version of the model	P_3D_	1100 Pa	Based on WSR
Pressure drop in 2D version of the model	P_2D_	615 Pa	Based on WSR

**Brinkman equations:** The Brinkman approach was used to calculate the flows in a vessel with a thrombus [47].

We used the following set of equations:


$$\left(\frac{\rho }{\;\epsilon }\right)*\frac{\partial 𝐮}{\partial \text{t}}+\;\left(\frac{\rho }{\;\epsilon }\right)\left(𝐮\nabla \right)\left(\frac{𝐮}{\epsilon }\right)=\nabla [-\text{pI}+\text{K}]-\mu \kappa^{-\text{1}}𝐮,$$ (2)

ρ∇𝐮=0, (3)

$$\text{K}=\left(\frac{\mu }{\epsilon }\right)\left(\nabla 𝐮+{\left(\nabla 𝐮\right)}^{T}-\left(\frac{\text{2}}{\text{3}}\right)\left(\nabla 𝐮\right)𝐈\right),$$ (4)

where ρ is the fluid density (1000 kg/m^3^), μ is the dynamic fluid viscosity, **u** is the flow velocity, p is the pressure, ϵ is the porosity, κ is the hydraulic permeability, **I** is the identity (unity) tensor.

The vessel walls and the surface of the injury site zone were considered as no-slip boundaries.

To solve Brinkman equations it is necessary to know the values of viscosity (μ), porosity (ϵ) and permeability (κ) at each moment in time and at each point in the vessel. In our model, the permeability value is completely determined by the porosity value (Eq. 1). Importantly, in our model local porosity and viscosity values depended on the platelet activation state, as discussed below.

**Platelet responses in the model:** Our model implies three types of platelet response to activation stimuli (Fig 1). The first response involves integrin activation which mediates platelet aggregation. In the model platelet aggregation process is described as formation of the region with high value of porosity that we term shell. This process is triggered by both thrombin and ADP. The second response is dense granule secretion that in our model is triggered by thrombin. The model describes this process as a localized flux of ADP molecules that depends on local thrombin concentration. The third response is platelet contraction that in our model is represented as localized transition to low porosity value. This region with this low porosity value we term the core. Importantly, the second and the third processes are considered to be correlated in the model: we imply that platelets that secreted some critical percentage of their dense granules, also participate in contraction process, hence lowering local porosity value. In order to compare simulation results with experimental data wherein core region is defined as thrombus area where platelet secreted their alpha-granules, we assume that the contracted platelets also secreted their alpha granules.

**Relationship between agonists concentration, platelet activation and local porosity:** In our model, thrombus formation is driven by thrombin and ADP. These activators play an essential role in thrombus formation in both laser-induced thrombosis and FeCl_3_-induced thrombosis [2,22].

To describe the formation of thrombus shell, we suggested that stable initial platelet attachment to the thrombus requires a significant level of agonist-induced αIIbβ3 integrin activation, and is only possible if the local concentration of thrombin or ADP exceeds threshold values. We considered that the thrombus shell zone instantaneously forms where agonist concentrations exceed these threshold values. Since the characteristic time of platelet integrin activation is about 1 second [11], which is significantly less than the characteristic time of thrombus growth in the laser-induced thrombosis model (about 1 minute [2]), we consider this assumption acceptable. In addition, for platelets activated only by ADP, we considered that if the local concentration of ADP falls below the threshold value, these platelets will be deactivated and will instantly detach from the thrombus, since ADP reversibly activates platelets [25]. In contrast, for thrombin-activated platelets, we assumed that platelets would remain activated and attached to the thrombus even if the thrombin concentration fell below a threshold value, since thrombin irreversibly activates platelets [26]. To describe this, we assumed that platelet integrin activation in response to thrombin depends on the maximum local concentration of thrombin over a time interval [0,t], where t is the current moment in time.

In agreement with the core-shell paradigm [2], we considered that the formation of the thrombus core zone is the result of platelet activation by high doses of thrombin (see below). Strong activation of platelets by thrombin is accompanied by granule release. Based on this, we postulated that the formation of the thrombus core zone occurs where the (normalized) platelet granule content (w) becomes less than the threshold value.

Taking all this into account, we used the following system of equations:


$$\epsilon =\;\left\{ \begin{array}{c} @c\text{1},\;\left[{\text{maxII}}_{\text{a}}\right]<{\text{v}}_{\text{1}}\text{ and }[\text{ADP}]<{\text{v}}_{\text{2}}\;\\ \text{shPor},\;\left[{\text{maxII}}_{\text{a}}\right]\geq {\text{v}}_{\text{1}}\text{ and w}>{\text{v}}_{\text{3}}\\ \text{shPor},\;[\text{ADP}]\geq {\text{v}}_{\text{2}}\text{ and w}>{\text{v}}_{\text{3}}\\ \text{corePor},\text{w }\leq {\text{v}}_{\text{3}} \end{array}\right.\;.$$ (5)

Where shPor is the thrombus shell porosity (0.65); corePor is the thrombus core porosity (0.45); [ADP] is the local concentration of ADP; [maxII_a_] is the maximal local concentration of thrombin in the interval [0,t], where t is the current moment in time; ${\text{v}}_{\text{1}}$ is the threshold of the platelet activation by thrombin (0.1 nM); $\;{\text{v}}_{\text{2}}$ is the threshold of the platelet activation by ADP (90 nM); w is the normalized platelet granule content; ${\text{v}}_{\text{3}}$ is the threshold value (0.368).

**Formation of the thrombus core zone:** As platelet granule secretion is an irreversible process and occurs in response to potent platelet activators – such as thrombin and collagen, it is usually considered as a marker of strong platelet activation. As *in vivo* data demonstrates that low porosity core region of thrombus possesses platelets that secreted their alpha-granules [2,4], we postulated that transition to low porosity value occurs only in the region where degree of granule release (w) is below some critical value. Platelet response to thrombin was described by sigmoidal function, based on *in vitro* data on thrombin-induced αIIbβ3 receptor activation and the secretion of dense granules [21,32] by mouse platelets. For simplicity, we assumed that in the wild type mice both platelet dense granule release and alpha granule release share the same kinetics.

To describe the kinetics of shell-to-core transformation we used the following equation:


$$\text{dw}/\text{dt}=-{\text{k}}_{\text{t}}({[\text{II}}_{\text{a}}])*\text{w},$$ (6)

Where w is the normalized platelet granule content, w (t = 0)=1; [II_a_] is the local thrombin concentration; k_t_([II_a_]) is rate of the process, which is the sigmoidal function describing platelet response to thrombin. We assumed that the transformation takes time and occurs when the normalized platelet granule content (w) falls below a threshold value ${\text{v}}_{\text{3}}$ = 1/e = 0.368.

Maximal rate of transformation K_max_ was set to the rate of dense granule release in response to high doses of thrombin [41]. Parameters of the sigmoid function describing dynamics of transformation were recovered from *in vitro* data on the dependence of total platelet ATP release on thrombin concentration [21] (see details in Supplemental Text C in S1 Text). Taking all this into account, we used the following equation:


$${\text{k}}_{\text{t}}\left({[\text{II}}_{\text{a}}]\right)=\left\{ {\text{K}}_{\text{max}}*(\begin{array}{c} \text{0},\left[{\text{II}}_{\text{a}}\right]\leq {\text{c}}_{\text{1}}\;\\ \text{6}*{\text{q}}^{\text{5}}-\text{15}*{\text{q}}^{\text{4}}+\text{10}*{\text{q}}^{\text{3}}),\;{\text{c}}_{\text{2}}\;\geq {[\text{II}}_{\text{a}}]\geq \\ {\text{K}}_{\text{max}},{[\text{II}}_{\text{a}}]\geq {\text{c}}_{\text{2}}\; \end{array}\right.\;{\text{c}}_{\text{1}}$$ (7)

Where c_1_ = 0.4 nM, c_2_ = 0.8 nM, q=(([II_a_]-(c_1_ + c_2_)/2)/(c_2_-c_1_))+0.5, K_max_ is the maximal rate of the process (0.15 s^-1^).

Due to the sigmoid nature of the thrombin response, the equations Eq. 6, Eq. 7 guarantee that the transformation of the shell zone into the core zone will not occur where the thrombin concentration is too low. This is consistent with the existence of large thrombus shell and experimental data on thrombin activity in the thrombus [2,4].

Equations Eq. 6, Eq. 7 were used to describe the formation of the thrombus core in both wild-type and mice with Hermansky-Pudlak syndrome, since the thrombus core is formed in both cases [2,20].

Relationship between agonist concentration and local viscosity: In our model, we considered two fluids with different viscosity values - blood plasma inside the thrombus, and whole blood in the vessel. To introduce this into the model we used the following equations:


$$\mu =\;\left\{ \begin{array}{c} @l\begin{array}{c} @l\eta_{\text{1}},\epsilon \leq \text{shPor }\\ \eta_{\text{2}},\epsilon >\text{shPor},\\ \; \end{array} \end{array}\right.\;$$ (8)

where μ is the local viscosity, $\;\eta_{\text{1}}$ is the blood plasma viscosity (1.25 mPa ⋅ s), $\eta_{\text{2}}$ is the whole blood viscosity (3.4 mPa ⋅ s).

For implementation purposes, instead of equation Eq. 8 we used an equivalent equation in which the porosity ϵ was expressed based on the concentrations of the agonists (using Eq. 5):


$$\mu =\;\left\{ \begin{array}{c} @c\;\begin{array}{c} @c\eta_{\text{1}},[\text{ADP}]\geq {\text{v}}_{\text{2}}\text{ or }\left[\text{maxI}{\text{I}}_{\text{a}}\right]\geq {\text{v}}_{\text{1}}\text{ or w}\leq \;{\text{v}}_{\text{3}}\\ \eta_{\text{2}},\left[\text{maxI}{\text{I}}_{\text{a}}\right]<{\text{v}}_{\text{1}}\text{ and }[\text{ADP}]<{\text{v}}_{\text{2}}\text{ and w}>{\text{v}}_{\text{3}},\;\\ \; \end{array} \end{array}\right.\;$$ (9)

### Generation and transport of thrombin and ADP

#### Generation of thrombin.

In the cremaster model thrombin has the potential to be generated both extravascularly, where TF is present [3], and intravascularly on the injured endothelium [48]. Thrombin generation on the surface of activated platelets does not seem to play the major role in cremaster model [48], and was not considered in our model, in line with another recent work [30].

To describe thrombin generation we modified the boundary condition and introduced an inward time-dependent thrombin flux from a zone on the surface of the injury site (blue zone on Fig 2A). This approach allowed us to take into account extravascular sources of thrombin. We used the following equation:


$$\text{J}(\text{t})=\;{\text{J}}_{\text{max}}*\left(\text{1}-\text{exp}\left(-\frac{\text{t}}{{\text{t}}_{\text{0}}}\right)\right),\;$$ (10)

Where t_0_ is the characteristic time of thrombin generation (30 s), ${\text{J}}_{\text{max}}\;$ is equilibrium thrombin flux (12 pmol/(m^2^ ⋅ s)) (we will refer to it simply as ‘thrombin flux’). These parameters were calibrated based on the comparison of simulation results with experimental videos of thrombus formation (S1 Fig).

We tested several options for the spatial location of the thrombin generation zone (S2 Fig). Thrombin generation in the upstream half of the injury site zone showed better agreement with experimental data compared to other alternatives (see Supplemental Text A in S1 Text).

We also performed additional simulations with a different dynamics of thrombin generation, see details in Supplemental Text A in S1 Text.

#### Inactivation of thrombin.

Thrombin is inactivated by several plasma proteins. The main pathway consists of complex formation with antithrombin III, but several other inhibitors, such as α1 -antitrypsin and α2 -macroglobulin, are active as well [49].

Computation of the constant of thrombin inactivation was performed based on data from the detailed computational model of coagulation by Dashkevich and colleagues [40]. We used the following equation:


$$\frac{\text{d }[\text{IIa}]}{\text{dt}}=-{\text{k}}_{\text{i}}*[\text{IIa}]$$ (11)

where [IIa]- local thrombin concentration, ${\text{k}}_{\text{i}}=\;$0.025 s^-1^. Details are described in Supplemental Text C in S1 Text.

#### Thrombin transport equation.

To describe the transport of thrombin, we used the following convection-diffusion equation, which takes into account the inactivation of thrombin by blood plasma inhibitors (Eq. 11):


$$\frac{\partial [{\text{II}}_{\text{a}}]}{\partial \text{t}}={\text{D}}_{\text{IIa}}*\Delta \left[{\text{II}}_{\text{a}}\right]-𝐮*\nabla \left[{\text{II}}_{\text{a}}\right]-{\text{k}}_{\text{i}}*\left[\text{I}{\text{I}}_{\text{a}}\right],$$ (12)

Where D_IIa_ is the thrombin diffusion coefficient, [IIa] is the local thrombin concentration. In the basic version of the model, thrombin diffusion coefficient did not depend on porosity. Computations with porosity-dependent thrombin diffusion coefficient are discussed in Supplemental Text A in S1 Text.

#### ADP release model.

The secretion of cargo molecules from platelet granules in response to activation is well described as a first-order reaction:


$$\frac{\text{dN}}{\text{dt}}=-{\text{k}}_{\text{ex}}*\text{N},$$ (13)

where N is the number of cargo molecules remaining in the platelet at time t, k_ex_ is the rate of secretion which depends on the cargo molecule, potency and dosage of the platelet agonist [41].

In out continuum approach, we used the following form of this equation:


$${\text{dN}}_{\text{ADP}}/\text{dt}=-{\text{k}}_{\text{ex}}*{\text{N}}_{\text{ADP}},$$ (14)

Where N_ADP_ is the local concentration of releasable ADP in platelet (amount of ADP in platelet dense granules divided by mean platelet volume).

In our model, we considered only thrombin-induced platelet secretion, in line with our previous study [19]. The dependence of the ADP secretion rate k_ex_ on the thrombin concentration was described by a sigmoid function. We used the same sigmoidal function describing platelet response to thrombin that we used to describe the process of thrombus core formation (see details in Supplemental Text C in S1 Text). Maximal rate of secretion K_max_ was set to the rate of dense granule release in response to high doses of thrombin [41]. Taking all this into account, we used the following equation:


$${\text{k}}_{\text{ex}}\left({[\text{II}}_{\text{a}}]\right)=\left\{ {\text{K}}_{\text{max}}*(\begin{array}{c} \text{0},\left[{\text{II}}_{\text{a}}\right]\leq {\text{c}}_{\text{1}}\;\\ \text{6}*{\text{q}}^{\text{5}}-\text{15}*{\text{q}}^{\text{4}}+\text{10}*{\text{q}}^{\text{3}}),\;{\text{c}}_{\text{2}}\;\geq {[\text{II}}_{\text{a}}]\geq \\ {\text{K}}_{\text{max}},{[\text{II}}_{\text{a}}]\geq {\text{c}}_{\text{2}}\; \end{array}\right.\;{\text{c}}_{\text{1}}$$ (15)

Where c_1_ = 0.4 nM, c_2_ = 0.8 nM, q=(([II_a_]-(c_1_ + c_2_)/2)/(c_2_-c_1_))+0.5, K_max_ is the maximal rate of secretion (0.15 s^-1^).

In our continuum approach, volumetric flux of ADP ${\text{J}}_{\text{ADP}}$ was described using the following equation:


$${\text{J}}_{\text{ADP}}={\text{N}}_{\text{ADP}}*(\text{1}-\epsilon )*{\text{k}}_{\text{ex}}([{\text{II}}_{\text{a}}])\;$$ (16)

Where ϵ is the local porosity value, and the factor (1−ϵ) corresponded to the fraction of the local volume occupied by platelets.

No-flux boundary condition was applied for ADP on the vessel walls and the surface of the injury site zone.

In our model, kinetics of thrombin-induced granule release (Eq. 15) is identical to the kinetics of thrombin-induced transformation of thrombus shell zone into thrombus core zone (Eq. 7). This feature of the model corresponds to the presence of two parallel processes - changes in porosity and granule secretion, which occur in the thrombus core on the same time scale [4].

#### ADP transport equation.

To describe the transport of ADP, we used the following convection-diffusion equation, which takes into account the ADP release from thrombin-activated platelets (Eq. 16):


$$\frac{\partial [\text{ADP}]}{\partial \text{t}}={\text{D}}_{\text{ADP}}*\Delta [\text{ADP}]-𝐮*\nabla [\text{ADP}]+{\text{N}}_{\text{ADP}}*(\text{1}-\epsilon )*{\text{k}}_{\text{ex}}\left(\left[{\text{II}}_{\text{a}}\right]\right),$$ (17)

Where D_ADP_ is the ADP diffusion coefficient, [ADP] is the local ADP concentration.

### Model implementation

#### Model equations.

Together, Brinkman equations (Eqs. 2–4), Kozeny-Carman equation (Eq. 1), porosity-concentration equations (Eq. 5), shell-to-core transformation equations (Eqs. 6, 7), viscosity-concentration equations (Eq. 9), thrombin transport equation (Eq. 12), thrombin generation equation (Eq. 10), ADP transport equation (Eq. 17), and platelet ADP content equation (Eq. 14) form the system of model equations. This system was solved numerically using Time-dependent Segregated solver in Comsol Multiphysics [35].

#### Initial conditions.

Initial velocity field and pressure field were the solution of stationary Navier-Stokes equations for the whole blood (described as a Newtonian incompressible fluid with dynamic viscosity 3.4 mPa ⋅ s) in the vessel without thrombus. Initial value of porosity was set to ϵ =1 everywhere, which corresponds to the vessel without thrombus. Initial value of viscosity was set to μ = 3.4 mPa ⋅ s everywhere (viscosity of the whole blood). Initial concentrations of thrombin and ADP were set to zero everywhere. Initial concentration of releasable ADP in platelet N_ADP_ was set to N_0_ = 1.15 mol/m^3^ everywhere in wild type mice; while it was set to N_0_ = 0 mol/m^3^ everywhere in mice with Hermansky-Pudlak syndrome. Initial value of the variable w describing normalized platelet granule content was set to w_0_ = 1 everywhere. These initial conditions are consistent with porosity-concentration equations (Eq. 5), viscosity-concentration equations (Eq. 9), and the rest of the model equations.

#### Computational Mesh.

To build the numerical mesh we utilized “Adaptive mesh refinement” feature in Comsol Multiphysics [35]. This approach adds mesh elements based on the error criterion to resolve those areas where the error is large, and allows to update mesh when necessary. Since the error indicator function depended on the magnitudes of the thrombin and ADP gradients (see Supplemental Text C in S1 Text), it reached large values where these gradients were large, namely, inside the thrombus. As a result, the mesh was significantly denser inside the thrombus, which made it possible to obtain accurate agonist profiles, which is crucial for model behavior. The numerical mesh consisted from about 750 000 elements and was updated every 2 seconds of the model time. The effect of the number of mesh elements on the simulation results was analyzed in Supplemental Text C in S1 Text (S10 Fig).

#### Simulation details and data processing.

Each simulation lasted 130 seconds of the biological time. The model predictions were compared to 3 experimental videos from 2 papers [2,20]. For this purpose, we calculated the dynamics of the thrombus area and the area of the thrombus core - an approach widely used in the original articles to analyze the dynamics of thrombus formation [2,4,20]. The dynamics of the thrombus area and the thrombus core area in the experiment (that is, the area occupied by CD 41 positive platelets and P-selectin positive platelets) was calculated based on the analysis of frames of experimental videos using a Python script. The dynamics of the thrombus area and the thrombus core area in the model simulations were inferred based on the 3D data by 2D integration over a plane passing through the center of the injury site zone and the horizontal axis of the vessel. To calculate the dynamics of the thrombus area, the following expression A_t_ was integrated:


$$\begin{array}{c} {\text{A}}_{\text{t}}=\;\left\{ \begin{array}{c} @l\begin{array}{c} @l\text{1},\epsilon \leq \text{shPor}\\ \text{0},\epsilon >\text{shPor} \end{array} \end{array}\right.\;\;\;\;\;, \end{array}$$ (18)

To calculate the dynamics of the thrombus core area, the following expression A_c_ was integrated:


$$\begin{array}{c} {\text{A}}_{\text{c}}=\;\left\{ \begin{array}{c} @l\begin{array}{c} @l\text{1},\epsilon \leq \text{corePor}\\ \text{0},\epsilon >\text{corePor} \end{array} \end{array}\right.\;\;\;\;\;, \end{array}$$ (19)

### Choice of the parameter values

In total, our model had 39 parameters. There were 2 parameters related to thrombin generation: characteristic time of thrombin generation t_0_ (30 s) and equilibrium thrombin flux J_max_. In 3D version of the model both values were fitted by comparing results of model simulations with thrombus growth dynamics in a representative experimental video of laser-induced thrombosis [20].

For the thrombus structure, model had 2 parameters: thrombus shell porosity and thrombus core porosity. For the thrombus shell porosity, we used shPor = 0.65. For the thrombus core porosity, we used corePor = 0.45. These values were taken from *in vivo* data on mouse thrombi (Fig 4 from [2]).

Platelets were described by 2 parameters: mean platelet volume, and average platelet diameter. Value of mean mouse platelet volume MPV = 4 fl was taken from *in vitro* data [43]. This value was also used to estimate average platelet diameter.

The model had 2 key parameters that described platelet activation by agonists. For the value of the platelet activation threshold by thrombin, we used ${\text{v}}_{\text{1}}=\text{0}.\text{1}\text{ nM}$. This value is equal to experimentally measured EC50 value for thrombin-induced integrin αIIbβ3 activation in mouse platelets [32]. For the value of the platelet activation threshold by ADP, we used ${\text{v}}_{\text{2}}=\text{9}\text{0}\text{ nM}$. This value is significantly lower than the experimentally measured EC50 value 590 nM for ADP-induced αIIbβ3 activation in mouse platelets, but it is close to the ADP concentration 100 nM, which causes ~10% of the maximal platelet response [32].

Two diffusion coefficients were used to describe the transport of agonists. The diffusion coefficient of thrombin, D_IIa_ = 67 µm^2^/s, was estimated based on experimental value for bovine thrombin [38] corrected for blood plasma viscosity. The diffusion coefficient of ADP D_ADP_ = 237 µm^2^/s was taken from published data [39].

Secretion of ADP from thrombin-activated platelets was described by 5 parameters. For the maximal ADP secretion rate, we used K_max_ = 0.15 s^-1^. This value was reported for the secretion of serotonin, a marker of ADP-containing dense granules, in response to activation by high doses of thrombin in human platelets [41]. In our model, the dependence of secretion kinetics on thrombin concentration was described by a sigmoid function recovered from *in vitro* data [21]. The sigmoid was described by 2 parameters: lower threshold of thrombin concentration, c_1_ = 0.4 nM, which corresponds to zero secretion rate, and upper threshold of thrombin concentration c_2_ = 0.8 nM, which corresponds to maximal secretion rate. These values were fitted to describe the experimentally observed relationship between total ATP secretion from mouse platelet dense granules and thrombin concentration [21](see details in Supplemental Text C in S1 Text). For the total amount of releasable ADP in mouse platelet, we used ADP_0_ = 0.46 ∙ 10^-8^ nmol. This value corresponds to the total amount of ADP in a mouse platelet [42], which means that it may be overestimated. Based on this value we calculated concentration of releasable ADP in mouse platelet N_0_ as the ratio between the total amount of releasable ADP and the platelet volume.

Transformation of shell zone into core zone was described by 4 parameters, 3 of which (the maximal rate of transformation and parameters of the sigmoid describing response to thrombin) were also used for ADP release model and they were described above. The threshold of transformation of the shell platelet into core platelet was set to 1/e = 0.368. According to equations Eq. 6, Eq. 7 this means that minimal time of transformation equals 1/K_max_ = 6.67 s. Since in experiments core starts to form about 25 seconds after the injury [4] and platelet activation time is about 1 second [11], we suggest that such minimal time of transformation is in the realistic range.

Parameters related to model geometry are discussed in the corresponding sections.

### 2D model of thrombogenesis

To analyze FeCl_3_ –induced thrombosis in mouse carotid artery we used 2D version of the continuum model described above. The structure of the model and its parameters were the same as in the 3D case. The only changes were introduced to the model geometry, hydrodynamics, thrombin generation and simulation procedures as described below.

#### Model geometry and hydrodynamics.

The model geometry reproduced the geometry of mouse carotid artery [22,23]. The computational domain consisted of a rectangular vessel with a width of 0.5 mm, and a length of 42.5 mm and an injury site zone. Such vessel geometry has the diameter/length ratio of 0.0117, in line with geometry described in [36] to account for the correct hemodynamic conditions, as the constant pressure drop boundary condition was applied for this domain. The injury site zone was described as a segment with a length of 1 mm located on the vessel wall (Fig 5A). The pressure drop was 615 Pa to ensure a wall shear rate of 1000 s^-1^ in a vessel without thrombus.

#### Thrombin generation.

The thrombin generation zone coincided with the injury site zone (blue segment on Fig 5A). Thrombin generation was described with Eq. 10. The value of the equilibrium thrombin flux J_max_ was varied depending on the degree of injury induced by different FeCl_3_ concentrations (see the Results section).

#### Simulation details.

Each simulation continued until either the occlusion was reached or 600 seconds of biological time were computed. Occlusion was defined as a 10 000-fold drop in blood flow compared to the blood flow in a vessel without thrombus. The model predictions were compared with experimental data from 2 papers [22,23].

Model also used more sophisticated error indicator function for mesh generation, than the 3D model, because gradients of thrombin and ADP were much larger in simulation of thrombus formation in artery compared to simulations in arterioles. The details are described in Supplemental Text C in S1 Text.

### 2D axisymmetric model of thrombogenesis

To describe the axisymmetric 3D case for simulations of thrombus growth in response to the circular injury, we used 2D axisymmetric version of the 3D model described above. Model structure and model parameters were the same as in the 3D model. The only changes were related to model geometry, hydrodynamics and thrombin generation as discussed below.

#### Model geometry and hydrodynamics.

The 2D axisymmetric geometry is viewed as the intersection between the original axially symmetric 3D solid and the half plane ϕ = 0, r ≥ 0. The computational domain consisted of a rectangular vessel with a width of 18 microns (corresponding to the 3D vessel radius) and a length of 3060 microns and an injury site zone. The injury site zone was described as the difference between an ellipse with semi-axes of 13.5 and 6 microns and a rectangular vessel (S11 Fig). The center of the ellipse was located on the wall of the vessel. The pressure drop was 1100 Pa to ensure a wall shear rate of 1000 s^-1^ in a vessel without thrombus.

#### Thrombin generation.

In line with 3D case, thrombin generation zone was located in the upstream half of the injury site zone (blue segment on S15 Fig). Thrombin generation followed the equation Eq. 10. Value of the equilibrium thrombin flux J_max_ was the same as in 3D case simulations.

The research is carried out using the equipment of the shared research facilities of HPC computing resources at Lomonosov Moscow State University (Moscow, Russia) [50].

## Supporting information

S1 Text Supplemental material. (DOCX)

S1 Movie Simulation of thrombus growth in the laser-induced injury of mouse cremaster muscle arterioles. **2D slice.** Video shows dynamics of thrombus growth in 2D longitudinal slice of the vessel in a wild type mouse. Vessel lumen is brown, thrombus core is dark blue, thrombus shell is blue. Flow direction was from top to the bottom. Time is shown in seconds. Color bar shows values of porosity corresponding to the colors on the video frames.
(MP4)

S2 Movie Simulation of thrombus growth in the laser-induced injury of mouse cremaster muscle arterioles. **3D view.** Video shows 3D view of the growing thrombus in a wild type mouse. Thrombus core is dark blue, thrombus shell is blue. Flow direction was from top to the bottom. Time is shown in seconds. Color bar shows the values of porosity corresponding to the colors on the video frames.
(MP4)

S3 Movie Simulation of thrombus growth in the laser-induced injury of HPS mouse cremaster muscle arterioles. **2D slice.** Video shows dynamics of thrombus growth in 2D longitudinal slice of the vessel in mouse with Hermansky-Pudlak syndrome. Vessel lumen is brown, thrombus core is dark blue, thrombus shell is blue. Flow direction was from top to the bottom. Time is shown in seconds. Color bar shows values of porosity corresponding to the colors on the video frames.
(MP4)

S4 Movie Simulation of thrombus growth in the laser-induced injury of HPS mouse cremaster muscle arterioles. **3D view.** Video shows 3D view of the growing thrombus in mouse with Hermansky-Pudlak syndrome. Thrombus core is dark blue, thrombus shell is blue. Flow direction was from top to the bottom. Time is shown in seconds. Color bar shows values of porosity corresponding to the colors on the video frames.
(MP4)

S5 Movie Simulation of thrombus growth in FeCl_3_-induced injury of the mouse carotid artery. **Occlusive thrombus.** Simulation shows thrombus growth in a wild type mouse which ended in vessel occlusion. Maximal thrombin flux was 2 pmol/(m^2^ ⋅ s). Vessel lumen is brown, thrombus core is dark blue, thrombus shell is blue. Flow direction was from top to the bottom. Time is shown in seconds. Color bar shows the values of porosity corresponding to the colors on the video frames.
(MP4)

S6 Movie Simulation of thrombus growth in a FeCl_3_-induced injury of the wild type mouse carotid artery. **Flow dynamics in the occlusion scenario.** Simulation shows the dynamics of flow during thrombus growth in a wild type mouse. Thrombus growth ended up in a vessel occlusion. Maximal thrombin flux was 2 pmol/(m^2^ ⋅ s). Vessel lumen is brown, thrombus core is dark blue, while thrombus shell is blue. Flow direction was from top to the bottom. Time is shown in seconds. Color bar shows the values of velocity magnitude (units [m/s]) corresponding to the colors on the video frames. This is exactly the same simulation as shown on the S5 Video.
(MP4)

S7 Movie Simulation of thrombus growth in a FeCl_3_-induced injury of the mouse carotid artery. **Non-occlusive thrombus.** Simulation shows thrombus growth in a wild type mouse. No occlusion was observed during 600 seconds of simulation. Maximal thrombin flux was 1.5 pmol/(m^2^ ⋅ s). Vessel lumen is brown, thrombus core is dark blue, thrombus shell is blue. Flow direction was from top to the bottom. Time is shown in seconds. Color bar shows the values of porosity corresponding to the colors on the video frames.
(MP4)

S1 Fig The effect of characteristic time of thrombin generation, thrombin flux, and platelet sensitivity to ADP on the dynamics of thrombus formation *in silico.* Computations were performed using 3D version of the model in microcirculation. On **A), B), C)** red, blue and robin egg blue dots correspond to thrombus area; orange, green and light magenta dots correspond to thrombus core area. **A)** The effect of characteristic time of thrombin generation on the temporal dynamics of thrombus core area and thrombus area in the model simulation. Red dots and orange dots correspond to characteristic time 20 seconds, blue and green dots correspond to characteristic time 30 seconds, robin egg blue dots and light magenta dots correspond to characteristic time 40 seconds. **B)** The effect of thrombin flux on the temporal dynamics of thrombus core area and thrombus area in the model simulation. Red dots and orange dots correspond to thrombin flux 8 pmol/(m^2^·s), blue and green dots correspond to thrombin flux 12 pmol/(m^2^·s), robin egg blue dots and light magenta dots correspond to thrombin flux 20 pmol/(m^2^·s). **C)** The effect of threshold of ADP-induced platelet activation on the temporal dynamics of thrombus core area and thrombus area in the model simulation. Red dots and orange dots correspond to threshold value 50 nM, blue and green dots correspond to threshold value 90 nM, robin egg blue dots and light magenta dots correspond to threshold value 150 nM. **D),E),F),G)**- Images of thrombus in the model simulations (vessel lumen is brown, shell is blue, core is dark blue). Each image corresponds to the thrombus at the end of the simulation with altered value of thrombin flux (**D, E**) or threshold of ADP-induced platelet activation (**F, G**). Flow direction was from top to bottom. On each image color bar shows values of the porosity corresponding to colors on this image**. D)** Thrombin flux 20 pmol/(m^2^·s). **E)** Thrombin flux 8 pmol/(m^2^·s). **F)** Threshold of ADP-induced platelet activation 150 nM. **G)** Threshold of ADP-induced platelet activation 50 nM. Note that images are presented with different scale bars.
(TIF)

S2 Fig The effect of localization of thrombin generation zone on the dynamics of thrombus formation. Computations were performed using 3D version of the model in microcirculation. Simulations were compared with experimental data on laser-induced thrombosis in mouse cremaster arterioles from Meng and colleagues [11]. **A)-** tested thrombin generation scenarios. The thrombin generation zone is marked in blue. Flow direction from right to left. **B), C)**- thrombin generation in the upstream half of the injury site zone. **D), E)** –thrombin generation in the downstream half of the injury site zone. **F), G)**- thrombin generation from the whole injury site zone. On **B), D), F)**: Temporal dynamics of thrombus core area and thrombus area *in vivo* (data from Movie A [11], orange and red dots) and in the model simulation (green and blue dots). On **C), E,) G)**: Image of thrombus in the end of simulation (vessel lumen is brown, shell is blue, core is dark blue). Flow direction from top to bottom. On each image color bar shows values of porosity corresponding to colors on this image.
(TIF)

S3 Fig The comparison between simulations with reversible and irreversible platelet activation by thrombin. Computations were performed using 3D version of the model in microcirculation. **A), B), C)**- reversible platelet activation by thrombin. **D), E), F)**- irreversible platelet activation by thrombin. **A), D)-** Temporal dynamics of thrombus core area and thrombus area *in vivo* (data from Movie A [11], orange and red dots) and in the model simulation (green and blue dots). **B), C), E), F) -** Images of thrombus in the model simulations (vessel lumen is brown, shell is blue, core is dark blue). Flow direction was from top to bottom. On each image color bar shows values of the porosity corresponding to colors on this image.
(TIF)

S4 Fig Effect of thrombin inactivation by plasma inhibitors on the dynamics of thrombus formation. Temporal dynamics of thrombus core and shell *in vivo* (wild type mouse; data from Movie A (orange and red dots) from [11]); and in the model simulations (green and dark blue dots for simulations without thrombin inactivation; light magenta and robin egg blue dots for simulations with thrombin inactivation).
(TIF)

S5 Fig Effect of the hindered thrombin diffusion on the dynamics of thrombus formation. The dynamics of thrombus formation was calculated using two models that assumed different types of dependence of the thrombin diffusion coefficient on porosity. **A), B), C)** – computations were performed with model HD1. **D), E), F) -** computations were performed with model HD2, with significantly decreased thrombin diffusion coefficient in thrombus core. **A), D)** - Images of thrombus in the model simulations (vessel lumen is brown, shell is blue, core is dark blue). Flow direction was from top to bottom. On each image color bar shows values of the porosity corresponding to colors on this image. **B), E)** - profiles of thrombin diffusion coefficient. On each image color bar shows values of the thrombin diffusion coefficient corresponding to colors on this image. **C), F)** – profiles of thrombin. On each image color bar shows values of the thrombin concentration corresponding to colors on this image. **G)** - Temporal dynamics of thrombus in the model simulations. Red and orange dots- simulations were performed with basic model, described in the manuscript. Dark blue and green dots- simulations were performed with model HD1. Robin egg blue dots and light magenta - simulations were performed with model HD2.
(TIF)

S6 Fig The effect of modified dynamics of thrombin generation on the dynamics of thrombus formation. **A)** Temporal dynamics of thrombus in the model simulations. Red and orange dots- simulations were performed with basic model, described in the manuscript. Dark blue and green dots- simulations were performed with DTF model. **B)** Image of thrombus in the DTF model simulation (vessel lumen is brown, shell is blue, core is dark blue). Flow direction was from top to bottom. Color bar shows values of the porosity corresponding to colors on this image. **C)** The temporal dependence of the thrombin flux in the DTF model.
(TIF)

S7 Fig The comparison between the dynamics of thrombus formation in the 3D model simulation and *in vivo* experiments on laser-induced thrombosis in the wild type mice. Temporal dynamics of thrombus area *in vivo* (data from Video 2 from Stalker and colleagues [1], red dots) and in the model simulation (blue dots).
(TIF)

S8 Fig Dynamics of thrombus formation before and after the occlusion in 2D continuum model of FeCl3-induced thrombosis in mouse carotid artery. Simulation lasted 400 seconds, thrombin flux was 2 pmol/(m^2^ ⋅ s). **A), B) -** Images of thrombus in the model simulation (vessel lumen is brown, shell is blue, core is dark blue). Flow direction was from top to bottom. Color bar shows values of the porosity corresponding to colors on this image. **C)** Temporal dynamics of the thrombus area in the model simulation. **D)** Temporal dynamics of thrombus core area in the model simulation. **E)** Temporal dynamics of blood flow in the model simulation.
(TIF)

S9 Fig The temporal dependence of the thrombin flux in the 3D continuum model of thrombus formation in microcirculation. (TIF)

S10 Fig Temporal dynamics of maximal agonist concentrations in the 3D continuum model of thrombus formation in microcirculation. Maximal concentrations of thrombin and ADP inside the whole computational domain (i.e., in thrombus and vessel) were calculated at each time moment. Note significant decrease of ADP concentration by the second minute.
(TIF)

S11 Fig Temporal dynamics of the areas of thrombin-activated zone and ADP-activated zone in the 3D continuum model of thrombus formation in microcirculation. Temporal dynamics of thrombin-activated area (red dots), ADP-activated area (orange dots), and overall thrombus area (blue dots).
(TIF)

S12 Fig The dependence between the rate of dense granules secretion and local thrombin concentration used in the 3D and 2D versions of the continuum model of thrombus formation. (TIF)

S13 Fig The effect of computational mesh size on the dynamics of thrombus formation in the 3D continuum model in microcirculation. Temporal dynamics of thrombus core area and thrombus area in the model simulation with normal mesh (green and blue dots) and with coarse mesh (orange and red dots).
(TIF)

S14 Fig The comparison between shell area in model and experiment. Computations were performed using 3D continuum model in microcirculation. Temporal dynamics of thrombus shell area in the model simulation (orange dots) and *in vivo* experiment (red dots). Experimental data from Movie A from [11]. Shell area was calculated as the difference between thrombus area and thrombus core area.
(TIF)

S15 Fig Geometry of the 2D axisymmetric continuum model of thrombus formation in microcirculation. Model geometry. 2D axisymmetric computational domain consisted of a rectangle representing the vessel and an injury site zone. Vessel radius was 18 microns, vessel length was 3060 microns. Injury zone was represented as an ellipse with semi-axes of 13.5 and 6 microns. Center of the ellipse was located on the vessel wall. The zone of thrombin generation is marked in blue. Flow direction was from top to bottom. The dotted line shows the axis of the vessel (symmetry axis).
(TIF)

S16 Fig The outcome of the 2D simulation of thrombus formation in mouse carotid artery with low thrombin flux. Vessel lumen is green. No thrombus was formed during 600 seconds of the model simulation. Thrombin flux was 1 pmol/(m^2^ ⋅ s).
(TIF)
