# Supplementary material for: Thrombin activity confinement and dense granule release drive the dynamics of arterial thrombus
Source: PLoS Comput Biol. 2026 Mar 20;22(3):e1014062. doi: 10.1371/journal.pcbi.1014062 (PMC13004377; doi:10.1371/journal.pcbi.1014062)
Supplement: S1 Text — (DOCX) [file pcbi.1014062.s001.docx]

**Supplemental Text A. General model behavior and robustness**

To test the model behavior and robustness we varied several parameters which describe thrombin generation and platelet response to thrombin and ADP. For thrombin generation, we varied characteristic time of thrombin generation, thrombin flux and localization of thrombin generation zone, as well as the shape of thrombin generation curve. For platelet response to thrombin, we varied type of platelet activation by thrombin through switching between irreversible and reversible platelet activation. For platelet response to ADP, we varied threshold of ADP-induced platelet activation. We also tested the effect of thrombin inactivation by blood plasma inhibitors and the effect of hindered thrombin diffusion in thrombus core on the dynamics of thrombus formation. All these simulations were performed in 3D version of the model in microcirculation. Results of the simulations are discussed below.

*Effect of characteristic time of thrombin generation and thrombin flux*

Changing the characteristic time of thrombin generation 2-fold had moderate quantitative effect on the overall dynamics of thrombus formation and its size (S1A Fig). However, the qualitative dynamics of thrombus formation remained unchanged. We conclude that the dynamics of thrombus formation is robust to changes in this parameter.

Changing the thrombin flux 2.5-fold had a major effect on thrombus size (S1B Fig, S1D Fig, S1E Fig). However, across the entire range of thrombin flux values ​​tested, the thrombus remained non-occlusive (S1D Fig, S1E Fig). We conclude that in the model thrombus size is sensitive to thrombin flux, while overall thrombus growth scenario is robust.

Intermediate values ​​of thrombin flux (12 pmol / (m^2^ · s)) and characteristic time of thrombin generation (30 s) allowed to obtain good agreement between the model calculations and the experimental results. These values ​​were used in the calculations presented in the paper.

*Localization of thrombin generation zone*

We tested 3 scenarios of localization of the thrombin production zone - in the upstream half of the injury zone, in the downstream half of the injury zone, and on the entire surface of the injury zone (S2A Fig). The thrombin flux value was fitted for each scenario separately, based on the thrombus core area value at the end of the simulation.

Changing the localization of thrombin generation zone had moderate effect on thrombus size and dynamics of thrombus formation (S2 Fig). Among all 3 tested scenarios, the localization of the thrombin generation zone in the upstream half of the injury zone showed the best similarity with the experimental data. It reproduced both the general dynamics of thrombus formation and thrombus core growth, as well as the experimental localization of the thrombus core, effectively filling the injury site zone (S2B Fig, S2C Fig). Second scenario, in which thrombin generation occurred in the downstream half of the injury zone, did not reproduce the localization of the thrombus core zone, which in this scenario was located only in the downstream half of the injury zone (S2D Fig, S2E Fig). Third scenario, in which the generation of thrombin occurred over the entire surface of the damaged zone, did not reproduce the experimental dynamics of thrombus core growth, predicting too long a delay time for thrombus core growth (S2F Fig).

We conclude that thrombin generation in the upstream half of the injury zone showed better agreement with the experiment compared to other tested scenarios. This scenario was used in the calculations presented in the paper.

*Irreversible vs reversible thrombin-induced platelet activation*

To test how the type of platelet activation by thrombin affects the calculation results, we ran two simulations assuming that thrombin activates platelets either reversibly or irreversibly. (S3 Fig).

The type of platelet activation by thrombin had a major effect on the dynamics of thrombus formation. In the scenario with reversible thrombin activation of platelets (S3A Fig, S3B Fig, S3C Fig), the residual thrombus size dropped significantly, which contradicts the experimental data. In contrast, in the scenario with irreversible thrombin-induced platelet activation (S3D Fig, S3E Fig, S3F Fig), the residual thrombus size dropped slightly compared to the maximum, and the dynamics of thrombus formation reproduced the experimental data.

We conclude that irreversible platelet activation by thrombin is essential for maintaining the large residual thrombus size in the model simulations. This scenario was used in the calculations presented in the paper.

*Effect of the platelet sensitivity to ADP*

Increasing the threshold of ADP-induced platelet activation 3-fold had moderate effect on thrombus size, but did not affect the thrombus core size (S1C Fig, S1F Fig, S1G Fig). However, the qualitative 3-stage dynamics of thrombus formation remained unchanged. We conclude that the dynamics of thrombus formation is robust to changes in this parameter.

Importantly, this result is in qualitative agreement with experimental observations by Stalker and colleagues (see Figure 5B and Figure 6D from [1]). They varied platelet sensitivity to ADP using either direct-acting P2Y12 antagonist, cangrelor, or genetically modified mice with altered platelet response downstream of P2Y12 receptor. In both cases, they observed a significant effect on the area of the thrombus shell, but not on the area of the thrombus core.

*Effect of thrombin inactivation by blood plasma inhibitors*

To test the effect of thrombin inactivation by plasma inhibitors we performed additional simulation where thrombin inactivation was turned off (S4 Fig). Turning off thrombin inactivation resulted in 2.5% increase of the overall thrombus area, and 3.6% increase of the thrombus core area at the end of the simulation (130 seconds). We conclude that thrombin inactivation by plasma inhibitors does not have significant effect on the dynamics of thrombus formation in the model simulations. Still, for the simulations presented in the paper, we used a scenario in which thrombin inactivation was taken into account.

*Effect of hindered thrombin diffusion*

Some studies [2],[3],[4] suggested that a significant decrease in the diffusion coefficient of the key molecules in the thrombus core could significantly affect the dynamics of thrombus formation. To test this effect we performed additional simulations using two versions of the basic model. In the first version, which we called HD1, we followed results of experimental paper [5] on the effect of porosity on diffusion coefficient in marine sediments. In this approach, thrombin diffusion coefficient D depended on porosity $\varepsilon$ via the following relationship:

| $D=\frac{D_{0}}{1+n\cdot(1-\varepsilon)}$ , |  | (S1) |
| --- | --- | --- |

Where n=3, D_0_- molecular diffusion coefficient of thrombin (6.7 $\cdot$10^-11^ m^2^/s). Computation gave 3.25 $\cdot$10^-11^ m^2^/s for thrombin diffusion coefficient in thrombus shell and 2.52$\cdot$ 10^-11^ m^2^/s for thrombin diffusion coefficient in thrombus core.

In the second model, which we called HD2, thrombin diffusion in thrombus shell still followed Eq. S1., but thrombin diffusion in thrombus core was additionally decreased 10 times, which gave the value 2.52 $\cdot$10^-12^ m^2^/s.

Computations with model HD1 showed moderate positive effect on thrombus size and thrombus core size, compared with computations using basic model (S5A Fig, S5G Fig). This resulted from the increased thrombin concentration in thrombus (S5C Fig). Computations with model HD2 showed small positive effect on thrombus size and thrombus core size (S5D Fig, S5G Fig).

We conclude that hindered thrombin diffusion has moderate quantitative effect on the dynamics of thrombus formation. However, it does not change this dynamics qualitatively.

*The effect of modified dynamics of thrombin generation on the dynamics of thrombus formation*

Some studies [6] suggested that thrombin generation in thrombus can decrease on a timescale of several minutes. To test this effect we performed additional simulation in which we changed the dynamics of thrombin generation. Instead of equation Eq. 10, used in the manuscript, we used the following expression for thrombin flux from the injury site:

| $J\left( t \right)=J_{\max}*\left( \frac{t}{t_{0}} \right)*exp(1-t/t_{0})$ , |  | (S2) |
| --- | --- | --- |

Where t_0_=60 s –characteristic time of the process, J_max_ – maximal thrombin flux (12 pmol/(m^2^⋅s)). Value of J_max_ was equal to the value of maximal thrombin flux used in the basic model described in the manuscript.

We called this model DTF (“decreasing thrombin flux”). In this model thrombin flux decreases about 30% at the end of simulation (130 s) compared to its maximal value (S6C Fig).

Computations using this model showed moderate positive effect on the dynamics of thrombus area, and small effect on the dynamics of thrombus core area (see S6 Fig). This was due to the increased flux of thrombin during first minute of thrombus growth (compare S6C Fig and S9 Fig). Decrease of the thrombin flux during the second minute of the simulation did not have significant effect on thrombus dynamics due to irreversible platelet activation by thrombin, considered in the model.

We conclude that the fast decrease of thrombin generation does not qualitatively affect dynamics of thrombus formation in the model simulations.

**Supplemental Text B. Additional data**

*Additional comparison between experimental data on the laser-induced thrombosis and 3D continuum model simulations of thrombus formation.*

To better test the model, we compared our calculations with the dynamics of thrombus formation in another experimental video of laser-induced thrombosis in microvessels from the article by Stalker and colleagues [1] (S7 Fig). The geometry of the vessel and injury zone in this video was similar to the geometry of the model (Table A), so for comparison we used a basic version of the 3D continuum model without modifications. Experimental video did not contain the data on thrombus core platelets (P-selectin positive platelets), so we only compared the thrombus area in the model and experiment. The dynamics of thrombus formation in the model showed good similarity with the experimental dynamics.

*Thrombus dynamics after occlusion in macrocirculation*

To analyze model predictions for the post-occlusion stage we performed additional simulation of FeCl_3_-induced thrombosis using 2D continuum model. Model predicted that both thrombus area and thrombus core area continued to grow after vessel occlusion (S8 Fig). Importantly, model does not explicitly consider platelet flux to the surface of thrombus, which changes drastically upon occlusion due to decrease of flow-mediated platelet flux to the thrombus surface. Hence, we believe that this conclusion is unreliable, and the model should be applied with caution for such kind of scenarios when flow rate is decreased significantly.

We want to note that in this simulation occlusion occurred later than in the simulation with similar injury size and thrombin flux presented in the manuscript (326 seconds against 248 seconds). The difference between these simulations is the distance between injury site zone and the vessel outlet (9.5 mm against 4.5 mm). Increased distance probably affected technical procedure of adaptive meshing, and as a result- slowed down the dynamics of thrombus formation.

**Supplemental Text C. Technical details**

In this chapter we present technical details related to the computation of parameters of the kinetics of thrombin-induced granule release and computation of the constant of thrombin inactivation. We also present several details related to computational meshing, including mesh sensitivity analysis and detailed description of the error indicator function used for adaptive mesh refinement.

*The kinetics of thrombin-induced granule release*

*1. Fitting details*

In our model, the kinetics of granule release from activated platelets depends only on the thrombin concentration. This kinetics is described with sigmoid function (see Eq. 15, and S12 Fig). This function depends on 2 threshold parameters: c_1_ and c_2_. These parameters were fitted based on the *in vitro* data on dense granule secretion [7]. Figure 3A from [7] describes the percentage of ATP secretion at different doses of thrombin.

Figure legend reports that for the wild type mouse platelets 95% confidence interval for thrombin EC_50_  value was in range 0.068-0.086 units/ml, which gives estimation of EC_50_ 0.077 units/ml, or 0.616 nM (we considered that 1 unit/ml=8 nM). Next, figure legend reports that platelet secretion in response to thrombin concentration 0.06 units/ml (0.48 nM) was 10.3±3.7 % from maximal.

Based on these data we estimated values c_1_ and c_2_ as 0.4 nM and 0.8 nM respectively. Computations using Eq. 15 with these parameters gives thrombin EC50 value 0.6 nM. For secretion rate in response to thrombin concentration 0.06 units/ml, computation gives 5.8% from maximal. We suggest that such level of fitting is sufficiently accurate.

*2. Implementation*

The sigmoid function that was used in our model to describe the kinetics of thrombin-induced granule release (see Eq. 15) was implemented using built-in step function in Comsol Multiphysics. The following set of parameters was used: {Location: 0.6 [nM]; From :0 [1/s] ; To: 0.15[1/s]; Size of transition zone: 0.4 nM; Number of continuous derivatives: 2}. Our tests have shown that Step function in Comsol Multiphysics ({Location: 0.5, Size of transition zone :1}) is the implementation of smootherStep function [8] of the following kind:

| $\mathrm{smootherstep}\left( x \right)=S_{2}\left( x \right)= \left\{ \begin{aligned} 0,x\leq0 \\ 6\cdot x^{5}-15\cdot x^{4}+10\cdot x^{3}, 1\geq x\geq0 \\ 1, x\geq1 \end{aligned} \right.$. | (S3) |
| --- | --- |

This is exactly the same type of sigmoid function that was used in the model (see Eq. 15).

*Computation of the thrombin inactivation constant*

Thrombin is inactivated by several plasma proteins. The main pathway consists of

complex formation with antithrombin III, but several other inhibitors, such as

α_1_ -antitrypsin and α_2_ -macroglobulin, are active as well [9].

Computation of the constant of thrombin inactivation was performed using the computational model by Dashkevich and colleagues [10]. Equation S30 in Supplemental material from [10] gives the following rate of thrombin inactivation:

| $\frac{d [IIa]}{\mathrm{dt}}=-\left( h_{9}\cdot i_{1}+h_{11}\cdot i_{7}+h_{12}{\cdot i}_{10}+h_{13}\cdot i_{9} \right)\cdot\left[ II_{a} \right]-h_{10}\cdot i_{6}\cdot(\left[ \mathrm{II}_{a} \right]+\left[ \mathrm{II}_{\mathrm{as}} \right])$ , |  | (S4) |
| --- | --- | --- |
|  |  |  |

Where $i_{1}$ is the concentration of antithrombin III, $i_{7}$ is the concentration of α_1_-antitrypsin, $i_{10}$ is the concentration of protein C inhibitor, $i_{9}$ is the concentration of heparin cofactor II, $i_{6}$ is the concentration of α_2_-macroglobulin, $[II_{a}]$ is the concentration of free thrombin, $[II_{\mathrm{as}}]$ is the surface density of thrombin, h_9,_h_11_,h_12,_h_13,_h_10_  are the rate constants of the corresponding inhibition reactions. In this equation we neglected the last term from original equation S30 from [10], because it describes thrombin interaction with thrombomodulin. It is a surface reaction; for the sake of simplicity, we did not consider surface reactions in our computations. For the same reason we set [II_as_ ]=0 in Eq.S4.

Next, we suggested that consumption of plasma inhibitors due to their interaction with thrombin can be neglected, because concentrations of plasma inhibitors are much larger than typical thrombin concentration in our simulations, which is about 1-3 nM. Then Eq.S4 turns into first-order reaction

| $\frac{d [IIa]}{\mathrm{dt}}=-k_{i}{\cdot[II}_{a}]$  , | (S5) |
| --- | --- |

Where k_i_ is the constant of thrombin inactivation. Computations with the values of parameters taken from the same paper by Dashkevich and colleagues (see Table S2, Table S3 from Supplemental material from [10]) give k_i_=0.0261 s^-1^. We used the rounded value k_i_=0.025 s^-1^ for all the computations presented in the paper.

*Mesh sensitivity analysis*

To test the effect of the numerical mesh element number on model outputs computations we performed two simulations using 3D continuum model of thrombus formation in microvessels. The first simulation used mesh with coarse mesh element size (231 954 elements at the last mesh of the simulation) (S13 Fig). The second simulation used mesh with normal mesh element size, which contained approximately 3 times more elements (781 659 elements at the last mesh of simulation) (S13 Fig). Results of the simulations were qualitatively similar. We conclude that the results of the model calculations are robust to the number of mesh elements. Simulations with normal mesh size are presented in the paper.

*Technical details on error indicator function in Adaptive mesh refinement*

In our simulations thrombus formation depended on the local concentration of agonists (thrombin and ADP), and vice versa. To improve the accuracy of computations we used the adaptive mesh refinement- computational approach which adds mesh elements based on an error criterion to resolve those areas where the error is large, which allows to improve performance of the computation [11].

Adaptive mesh refinement is controlled by the error indicator function. When computation is controlled by a single key variable c, Comsol Multiphysics user guide [12] recommend using the following expression for the error indicator function F:

$F\boldsymbol{=}\sqrt{\boldsymbol{(}{\frac{\partial\mathbf{c}}{\partial x})}^{\boldsymbol{2}}\boldsymbol{+(}{\frac{\partial\mathbf{c}}{\partial y})}^{\boldsymbol{2}}\boldsymbol{+(}{\frac{\partial\mathbf{c}}{\partial z})}^{\boldsymbol{2}}}\boldsymbol{,}$ (S6)

where c is the variable.

Our simulations of thrombus formation are controlled by 2 variables: thrombin concentration and ADP concentration. So, we modified equation Eq. S6 using thresholds of platelet activation by thrombin and ADP as normalizing factors:

$F=\sqrt{\boldsymbol{((}{\frac{\partial[\mathbf{I}\mathbf{I}_{\mathbf{a}}\boldsymbol{]}}{\partial x})}^{\boldsymbol{2}}\boldsymbol{+(}{\frac{\partial[\mathbf{I}\mathbf{I}_{\mathbf{a}}\boldsymbol{]}}{\partial y})}^{\boldsymbol{2}}\boldsymbol{+(}{\frac{\partial[\mathbf{I}\mathbf{I}_{\mathbf{a}}\boldsymbol{]}}{\partial z})}^{\boldsymbol{2}}\mathbf{)/}\boldsymbol{(}\boldsymbol{v}_{\boldsymbol{1}}^{\boldsymbol{2}}\mathbf{)+}\boldsymbol{((}{\frac{\partial[\mathbf{ADP]}}{\partial x})}^{\boldsymbol{2}}\boldsymbol{+(}{\frac{\partial[\mathbf{ADP]}}{\partial y})}^{\boldsymbol{2}}\boldsymbol{+(}{\frac{\partial[\mathbf{ADP]}}{\partial z})}^{\boldsymbol{2}}\boldsymbol{)/(}\boldsymbol{v}_{\boldsymbol{2}}^{\boldsymbol{2}}\mathbf{)}}$ (S7)

Where [II_a_ ] is the local thrombin concentration, [ADP] is the local ADP concentration, v_1_ is the threshold of thrombin-induced platelet activation, v_2_ is the threshold of ADP-induced platelet activation.

This expression was used in all the 3D simulations of thrombus formation in microvessels.

However, when we tried to use Eq. S7 (more accurately, its 2D version) for 2D simulations of thrombus formation in arteries, the simulation got stuck at some point and could not reach the convergence. We suggested that this problem was due to the structure of Eq. S7. For simulations in microvessels, ratio ‘maximal value/threshold’ was comparable for ADP and thrombin (35.6 and 17.5 times respectively). In contrast, for simulations in macrovessels this ratio was much higher for ADP than for thrombin (544 and 15.1 times respectively). As a result, error indicator function depended only on the ADP concentration, which caused problems with correct computations of thrombin profile and convergence issues. To solve these problems, we modified Eq. S7 and used the following 2D expression:

$F\mathbf{=}\sqrt{(({\frac{\partial\left[ II_{a} \right]}{\partial x})}^{2}+({\frac{\partial\left[ II_{a} \right]}{\partial y})}^{2})/([{\mathrm{II}_{a}]}^{2}+v_{1}^{2})+(({\frac{\partial\left[ \mathrm{ADP} \right]}{\partial x})}^{2}+({\frac{\partial\left[ \mathrm{ADP} \right]}{\partial y})}^{2})/([{ADP]}^{2}+v_{2}^{2})}$ (S8)

Where [II_a_] is the local thrombin concentration, [ADP] is the local ADP concentration, v_1_ is the threshold of thrombin-induced platelet activation, v_2_ is the threshold of ADP-induced platelet activation.

This expression showed good performance and allowed successful computation of different scenarios of FeCl_3_-induced thrombus formation in mouse carotid artery presented in the paper.

**Supplemental Text D. Processing model data and experimental data.**

*Processing data in Comsol Multiphysics*

To calculate dynamics of thrombus area we used 2D integration of the following expression over computational domain:

$(br.epsilon\_p\leq0.65)$ (S9)

where br.epsilon_p is the local porosity value, calculated in Brinkman Equations module, 0.65 is the porosity of thrombus shell.

To calculate dynamics of thrombus core area we used 2D integration of the following expression over computational domain:

$(br.epsilon\_p\leq0.45)$ (S10)

where br.epsilon_p is the local porosity value, calculated in Brinkman Equations module, 0.45 is the porosity of thrombus core.

The following instruction can be used to perform such computation after the end of the simulation.

1. Build proper 2D dataset, corresponding to longitudinal slice: Results>> Datasets>>Cut Plane (Dataset: Study1/Refined Mesh Solution, Plane: yz plane)
2. Calculate dynamics of thrombus area: Derived values>>Integration>>Surface Integration (Selection: All domains, Expression:$br.epsilon\_p\leq0.65$ )>>Evaluate
3. Calculate dynamics of thrombus core area: Derived values>>Integration>>Surface Integration (Selection: All domains, Expression:$br.epsilon\_p\leq0.45$ )>>Evaluate

*Processing frames from experimental videos*

In total, we processed 3 experimental videos: 2 videos from the paper by Meng and colleagues [11], and 1 video from the paper by Stalker and colleagues [1]. To split video into frames we used library ffmpeg in Linux. We used the following algorithm:

1. Copy video named ‘video.mov’ into folder, open this folder, then paste

ffmpeg -i video.mov -q:v 1 -vf "select=not(mod(n\,3))" -vsync vfr output_%04d.jpg

1. After that sequence of video frames appears in this folder. Copy this folder and use it to process images using web tools and python script.

For primary video analysis we used a web tool [13]. This tool was used to measure the number of pixels in the color bar and the geometry of the injury site (see Table A), as well as to estimate manually thresholds for pixel analyses (see below).

To process video frames we used custom script written in Python. Videos from two papers were processed separately, because they had different color structure. Videos from the paper by Meng and colleagues had red and yellow zones (for shell platelets and core platelets respectively). Video from the paper by Stalker and colleagues had red, blue and purple zones (for platelets without fibrin, fibrin without platelets, and fibrin with platelets respectively). All the videos also had grey zones (vessel, tissues) and white signs for time bar and scale bar.

To separate the colored zone from the gray zone we used threshold-like expression:

Abs (red-blue) + Abs (red-green) + Abs (green-blue) > threshold

Where red, green, and blue are the values of corresponding colors in the given pixel. Threshold values 50 and 80 were used for videos from the paper by Meng and colleagues and paper by Stalker and colleagues respectively.

Additional conditions of the same type were used to define thrombus zone, depending on the specific features of each video. For example special ‘green arrow condition’ was used to get rid of green arrow showing position of fibrin on Video 2 from paper by Stalker and colleagues [1]. Similar approach was used to define thrombus core zone.

At some experimental videos tiny thrombi formed downstream or upstream injury site zone. To get rid of them we used an additional algorithm. We calculated center mass of all points of thrombi defined by expressions described above, calculated the distance from these points to the center mass, sorted array of these distances, and analyzed differences between consecutive values in the sorted array. If difference between pair of values exceeded threshold value (which indicated that the next points in the array were not connected with the mass center), then we suggested that all these next points belonged to a different thrombus.

**Table A.** Geometry of experimental videos and model geometry. Values from experimental videos were obtained by measuring pixel coordinates of manually chosen points at the frames at 130th second. Due to the complex shape of the vessel and injury site reported values (especially the injury site depth) should be considered as approximate estimates.

|  | Vessel diameter (µm) | Injury site length (µm) | Injury site depth (µm) | Scale bar (number of pixels in 10 µm ) |
| --- | --- | --- | --- | --- |
| Video 2 from Stalker et al.  [1] | 29 | 22 | 4 | 31 |
| Movie A from Meng et al.  [11] | 36 | 28 | 6 | 20 |
| Movie D from Meng et al.  [11] | 29 | 26 | 4 | 20 |
| 3D continuum model | 36 | 27 | 6 |  |

**Supplemental References**

[1] T. J. Stalker *et al.*, ‘Hierarchical organization in the hemostatic response and its relationship to the platelet-signaling network’, *Blood*, vol. 121, no. 10, pp. 1875–1885, Mar. 2013, doi: 10.1182/blood-2012-09-457739.

[2] J. D. Welsh *et al.*, ‘A systems approach to hemostasis: 1. The interdependence of thrombus architecture and agonist movements in the gaps between platelets’, *Blood*, vol. 124, no. 11, pp. 1808–1815, Sep. 2014, doi: 10.1182/blood-2014-01-550335.

[3] M. Tomaiuolo, T. J. Stalker, J. D. Welsh, S. L. Diamond, T. Sinno, and L. F. Brass, ‘A systems approach to hemostasis: 2. Computational analysis of molecular transport in the thrombus microenvironment’, *Blood*, vol. 124, no. 11, pp. 1816–1823, Sep. 2014, doi: 10.1182/blood-2014-01-550343.

[4] K. Leiderman and A. L. Fogelson, ‘The influence of hindered transport on the development of platelet thrombi under flow’, *Bull. Math. Biol.*, vol. 75, no. 8, pp. 1255–1283, Aug. 2013, doi: 10.1007/s11538-012-9784-3.

[5] N. Iversen and B. B. Jørgensen, ‘Diffusion Coefficients of Sulfate and Methane in Marine Sediments: Influence of porosity’, *Geochim. Cosmochim. Acta*, vol. 57, pp. 571–578, 1993.

[6] Y. Lu, M. Y. Lee, S. Zhu, T. Sinno, and S. L. Diamond, ‘Multiscale simulation of thrombus growth and vessel occlusion triggered by collagen/tissue factor using a data-driven model of combinatorial platelet signalling’, *Math. Med. Biol. J. IMA*, vol. 34, no. 4, pp. 523–546, Dec. 2017, doi: 10.1093/imammb/dqw015.

[7] E. M. Golebiewska *et al.*, ‘Syntaxin 8 regulates platelet dense granule secretion, aggregation, and thrombus stability’, *J. Biol. Chem.*, vol. 290, no. 3, pp. 1536–1545, Jan. 2015, doi: 10.1074/jbc.M114.602615.

[8] ‘Smoothstep’, *Wikipedia*. Apr. 06, 2025. Accessed: Apr. 15, 2025. [Online]. Available: https://en.wikipedia.org/w/index.php?title=Smoothstep&oldid=1284255630

[9] H. Kessels, G. Willems, and H. C. Hemker, ‘Analysis of thrombin generation in plasma’, *Comput. Biol. Med.*, vol. 24, no. 4, pp. 277–288, Jul. 1994, doi: 10.1016/0010-4825(94)90024-8.

[10] N. M. Dashkevich *et al.*, ‘Thrombin activity propagates in space during blood coagulation as an excitation wave’, *Biophys. J.*, vol. 103, no. 10, pp. 2233–2240, Nov. 2012, doi: 10.1016/j.bpj.2012.10.011.

[11] R. Meng *et al.*, ‘Defective release of α granule and lysosome contents from platelets in mouse Hermansky-Pudlak syndrome models’, *Blood*, vol. 125, no. 10, pp. 1623–1632, Mar. 2015, doi: 10.1182/blood-2014-07-586727.

[12] ‘COMSOL Multiphysics’, *Wikipedia*. Nov. 19, 2024. Accessed: Apr. 15, 2025. [Online]. Available: https://en.wikipedia.org/w/index.php?title=COMSOL_Multiphysics&oldid=1258393905

[13] ‘Image inspection multi-tool - Pix Spy’. Accessed: Mar. 12, 2025. [Online]. Available: https://pixspy.com/
