# Supplementary figures and images for: Thrombin activity confinement and dense granule release drive the dynamics of arterial thrombus
Source: PLoS Comput Biol. 2026 Mar 20;22(3):e1014062. doi: 10.1371/journal.pcbi.1014062 (PMC13004377; doi:10.1371/journal.pcbi.1014062)

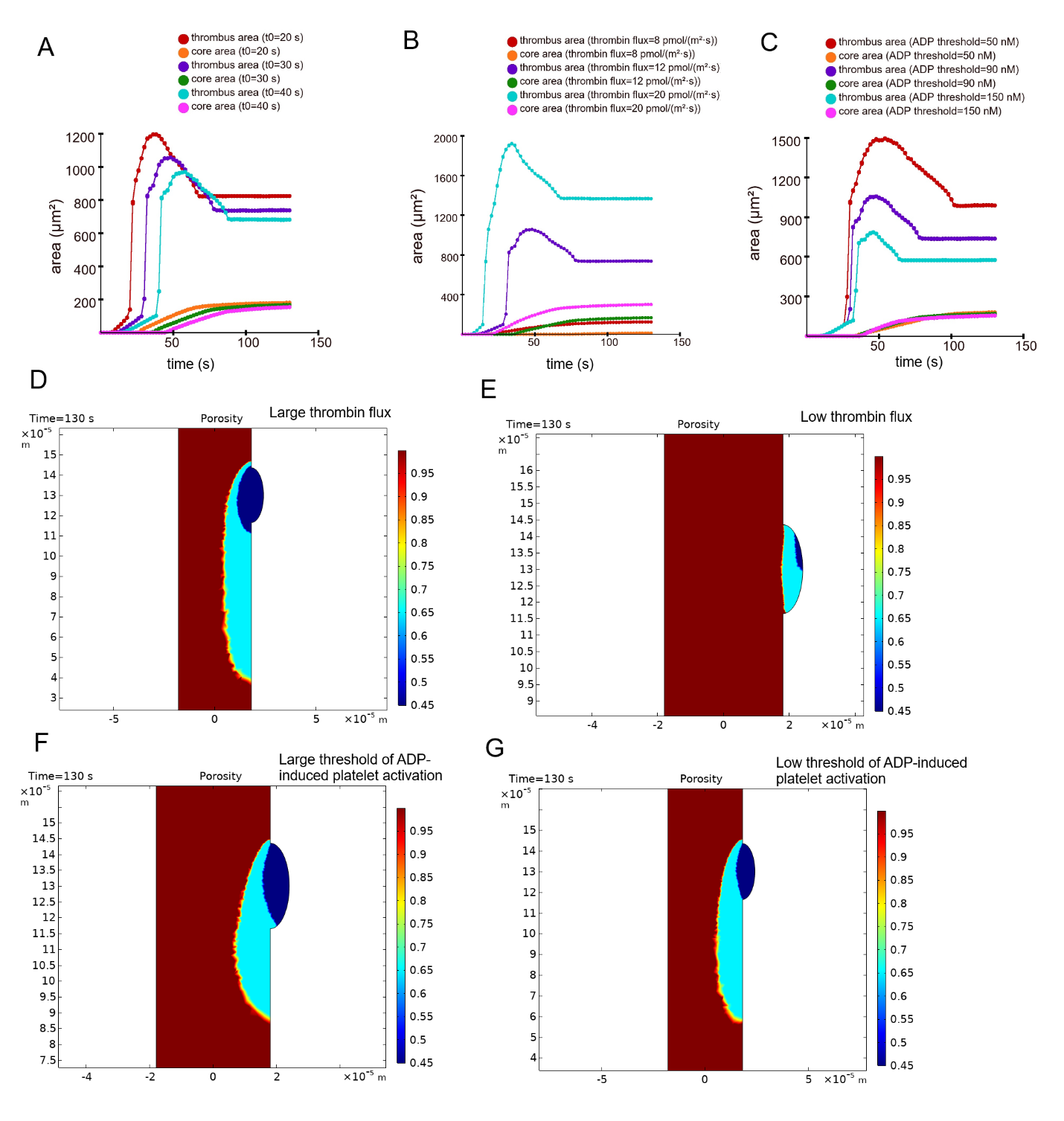

Supplement: S1 Fig — Computations were performed using 3D version of the model in microcirculation. On A), B), C) red, blue and robin egg blue dots correspond to thrombus area; orange, green and light magenta dots correspond to thrombus core area. A) The effect of characteristic time of thrombin generation on the temporal dynamics of thrombus core area and thrombus area in the model simulation. Red dots and orange dots correspond to characteristic time 20 seconds, blue and green dots correspond to characteristic time 30 seconds, robin egg blue dots and light magenta dots correspond to characteristic time 40 seconds. B) The effect of thrombin flux on the temporal dynamics of thrombus core area and thrombus area in the model simulation. Red dots and orange dots correspond to thrombin flux 8 pmol/(m2·s), blue and green dots correspond to thrombin flux 12 pmol/(m2·s), robin egg blue dots and light magenta dots correspond to thrombin flux 20 pmol/(m2·s). C) The effect of threshold of ADP-induced platelet activation on the temporal dynamics of thrombus core area and thrombus area in the model simulation. Red dots and orange dots correspond to threshold value 50 nM, blue and green dots correspond to threshold value 90 nM, robin egg blue dots and light magenta dots correspond to threshold value 150 nM. D),E),F),G)- Images of thrombus in the model simulations (vessel lumen is brown, shell is blue, core is dark blue). Each image corresponds to the thrombus at the end of the simulation with altered value of thrombin flux (D, E) or threshold of ADP-induced platelet activation (F, G). Flow direction was from top to bottom. On each image color bar shows values of the porosity corresponding to colors on this image. D) Thrombin flux 20 pmol/(m2·s). E) Thrombin flux 8 pmol/(m2·s). F) Threshold of ADP-induced platelet activation 150 nM. G) Threshold of ADP-induced platelet activation 50 nM. Note that images are presented with different scale bars. (TIF) [file pcbi.1014062.s009.tif]

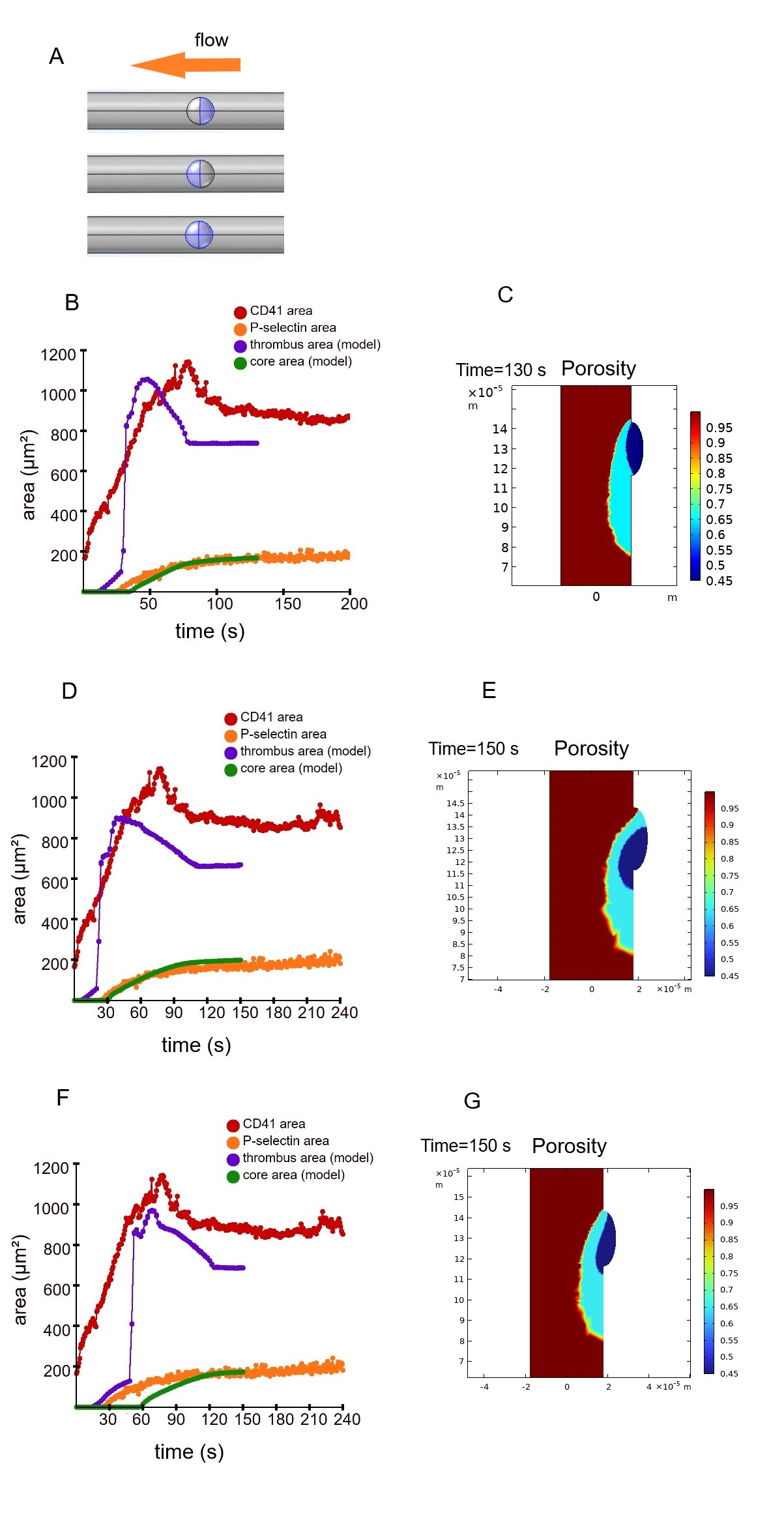

Supplement: S2 Fig — Computations were performed using 3D version of the model in microcirculation. Simulations were compared with experimental data on laser-induced thrombosis in mouse cremaster arterioles from Meng and colleagues [11]. A)- tested thrombin generation scenarios. The thrombin generation zone is marked in blue. Flow direction from right to left. B), C)- thrombin generation in the upstream half of the injury site zone. D), E) –thrombin generation in the downstream half of the injury site zone. F), G)- thrombin generation from the whole injury site zone. On B), D), F): Temporal dynamics of thrombus core area and thrombus area in vivo (data from Movie A [11], orange and red dots) and in the model simulation (green and blue dots). On C), E,) G): Image of thrombus in the end of simulation (vessel lumen is brown, shell is blue, core is dark blue). Flow direction from top to bottom. On each image color bar shows values of porosity corresponding to colors on this image. (TIF) [file pcbi.1014062.s010.tif]

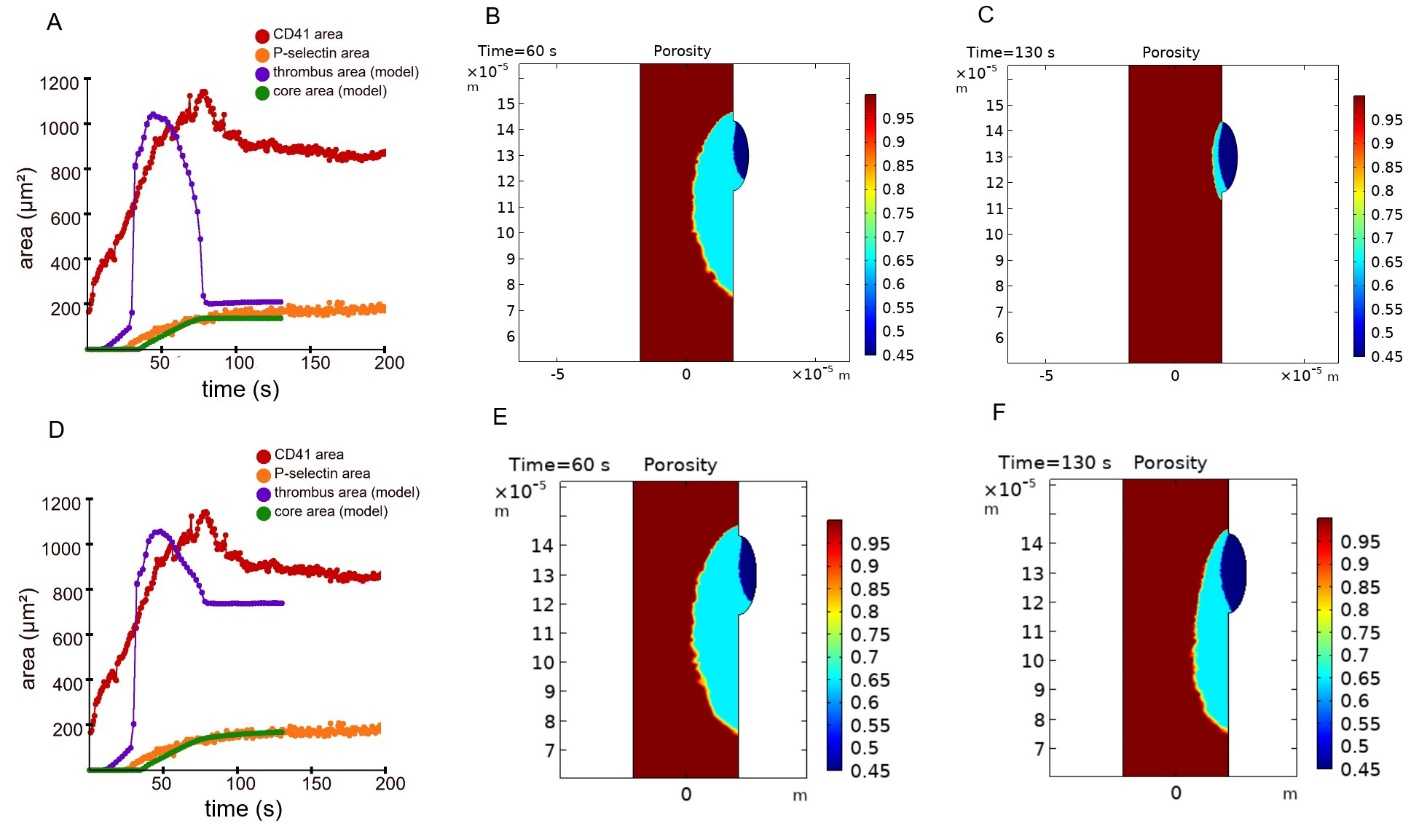

Supplement: S3 Fig — Computations were performed using 3D version of the model in microcirculation. A), B), C)- reversible platelet activation by thrombin. D), E), F)- irreversible platelet activation by thrombin. A), D)- Temporal dynamics of thrombus core area and thrombus area in vivo (data from Movie A [11], orange and red dots) and in the model simulation (green and blue dots). B), C), E), F) - Images of thrombus in the model simulations (vessel lumen is brown, shell is blue, core is dark blue). Flow direction was from top to bottom. On each image color bar shows values of the porosity corresponding to colors on this image. (TIF) [file pcbi.1014062.s011.tif]

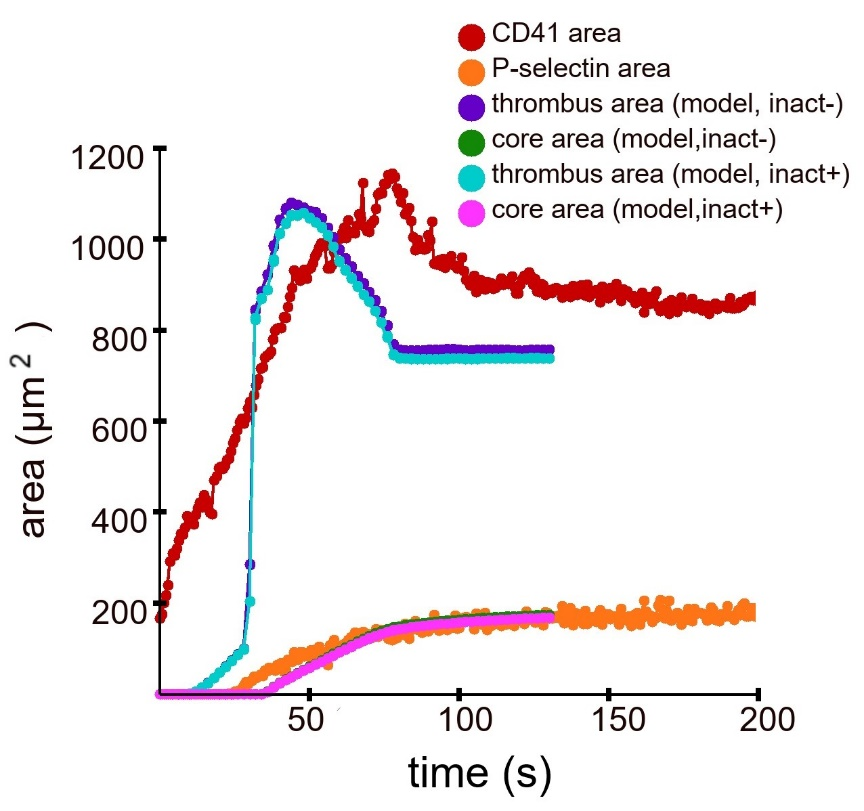

Supplement: S4 Fig — Temporal dynamics of thrombus core and shell in vivo (wild type mouse; data from Movie A (orange and red dots) from [11]); and in the model simulations (green and dark blue dots for simulations without thrombin inactivation; light magenta and robin egg blue dots for simulations with thrombin inactivation). (TIF) [file pcbi.1014062.s012.tif]

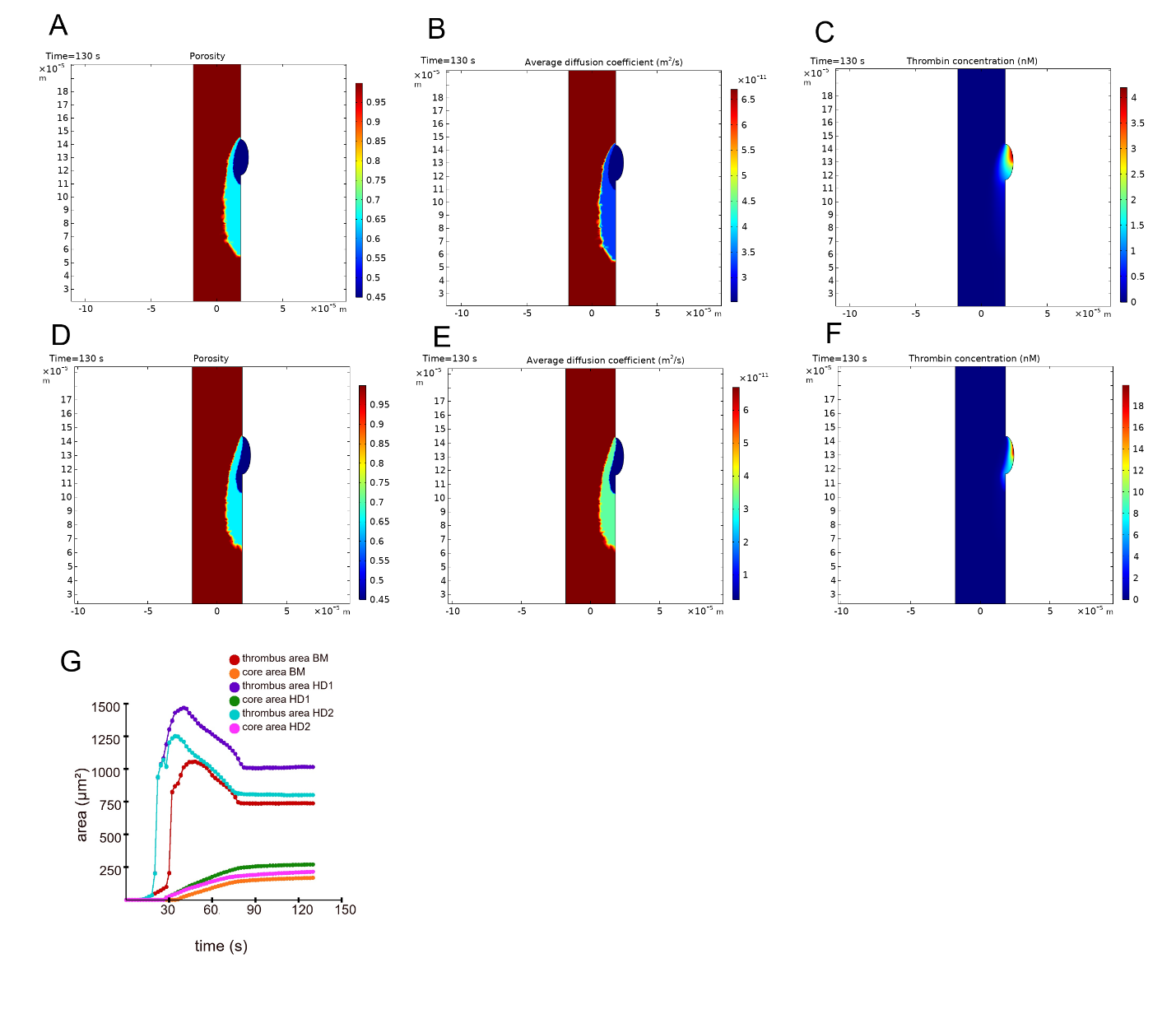

Supplement: S5 Fig — The dynamics of thrombus formation was calculated using two models that assumed different types of dependence of the thrombin diffusion coefficient on porosity. A), B), C) – computations were performed with model HD1. D), E), F) - computations were performed with model HD2, with significantly decreased thrombin diffusion coefficient in thrombus core. A), D) - Images of thrombus in the model simulations (vessel lumen is brown, shell is blue, core is dark blue). Flow direction was from top to bottom. On each image color bar shows values of the porosity corresponding to colors on this image. B), E) - profiles of thrombin diffusion coefficient. On each image color bar shows values of the thrombin diffusion coefficient corresponding to colors on this image. C), F) – profiles of thrombin. On each image color bar shows values of the thrombin concentration corresponding to colors on this image. G) - Temporal dynamics of thrombus in the model simulations. Red and orange dots- simulations were performed with basic model, described in the manuscript. Dark blue and green dots- simulations were performed with model HD1. Robin egg blue dots and light magenta - simulations were performed with model HD2. (TIF) [file pcbi.1014062.s013.tif]

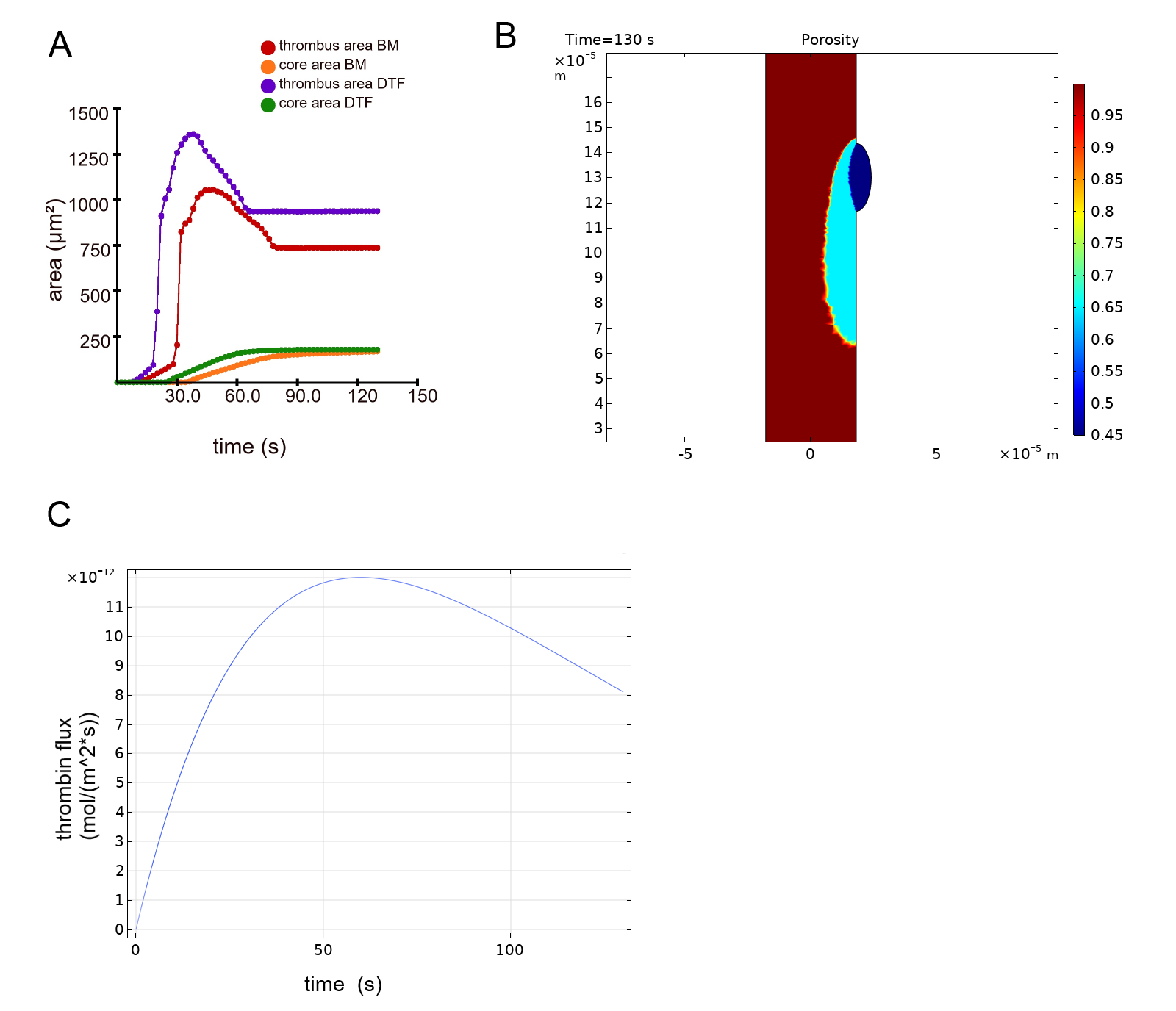

Supplement: S6 Fig — A) Temporal dynamics of thrombus in the model simulations. Red and orange dots- simulations were performed with basic model, described in the manuscript. Dark blue and green dots- simulations were performed with DTF model. B) Image of thrombus in the DTF model simulation (vessel lumen is brown, shell is blue, core is dark blue). Flow direction was from top to bottom. Color bar shows values of the porosity corresponding to colors on this image. C) The temporal dependence of the thrombin flux in the DTF model. (TIF) [file pcbi.1014062.s014.tif]

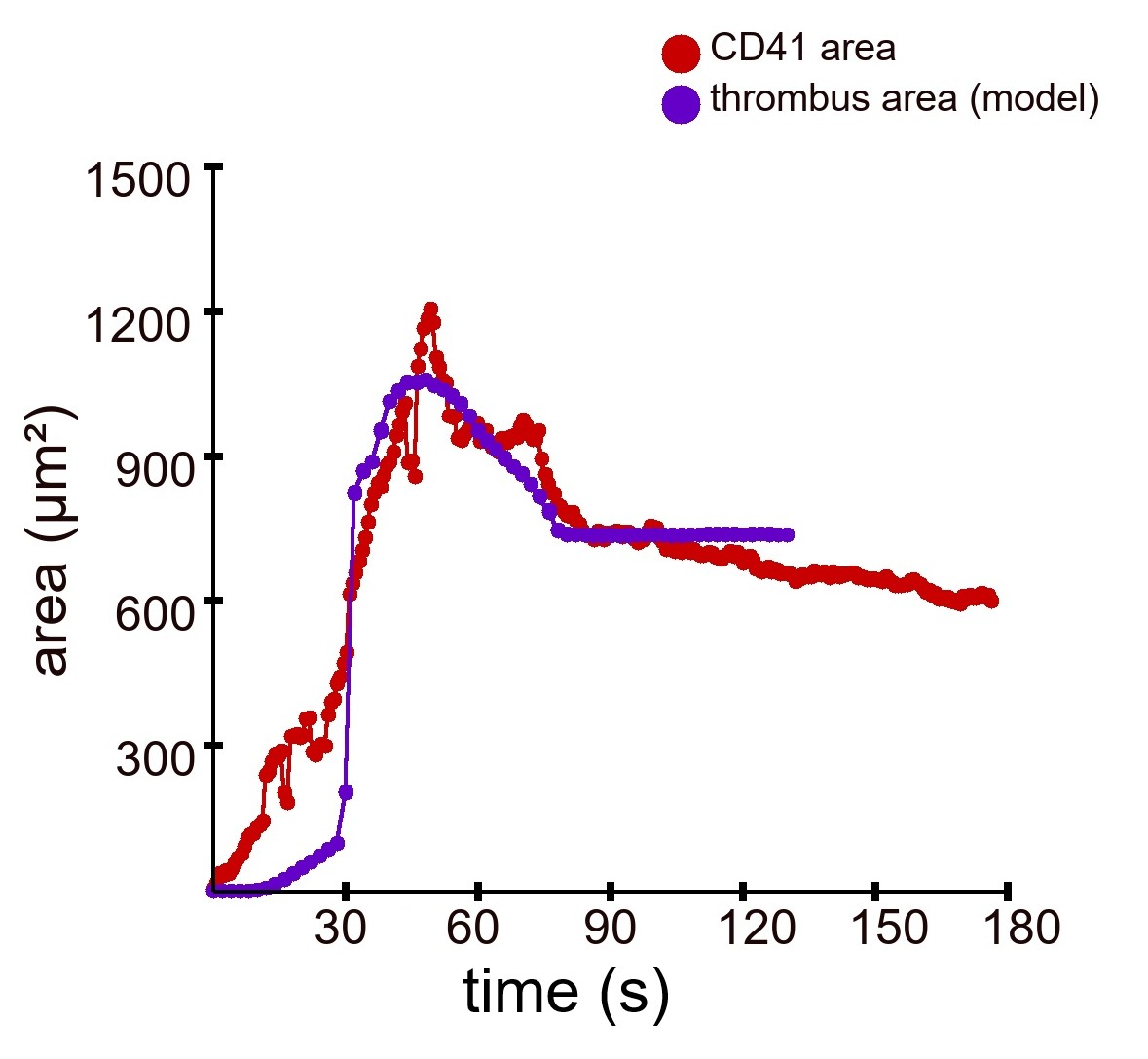

Supplement: S7 Fig — Temporal dynamics of thrombus area in vivo (data from Video 2 from Stalker and colleagues [1], red dots) and in the model simulation (blue dots). (TIF) [file pcbi.1014062.s015.tif]

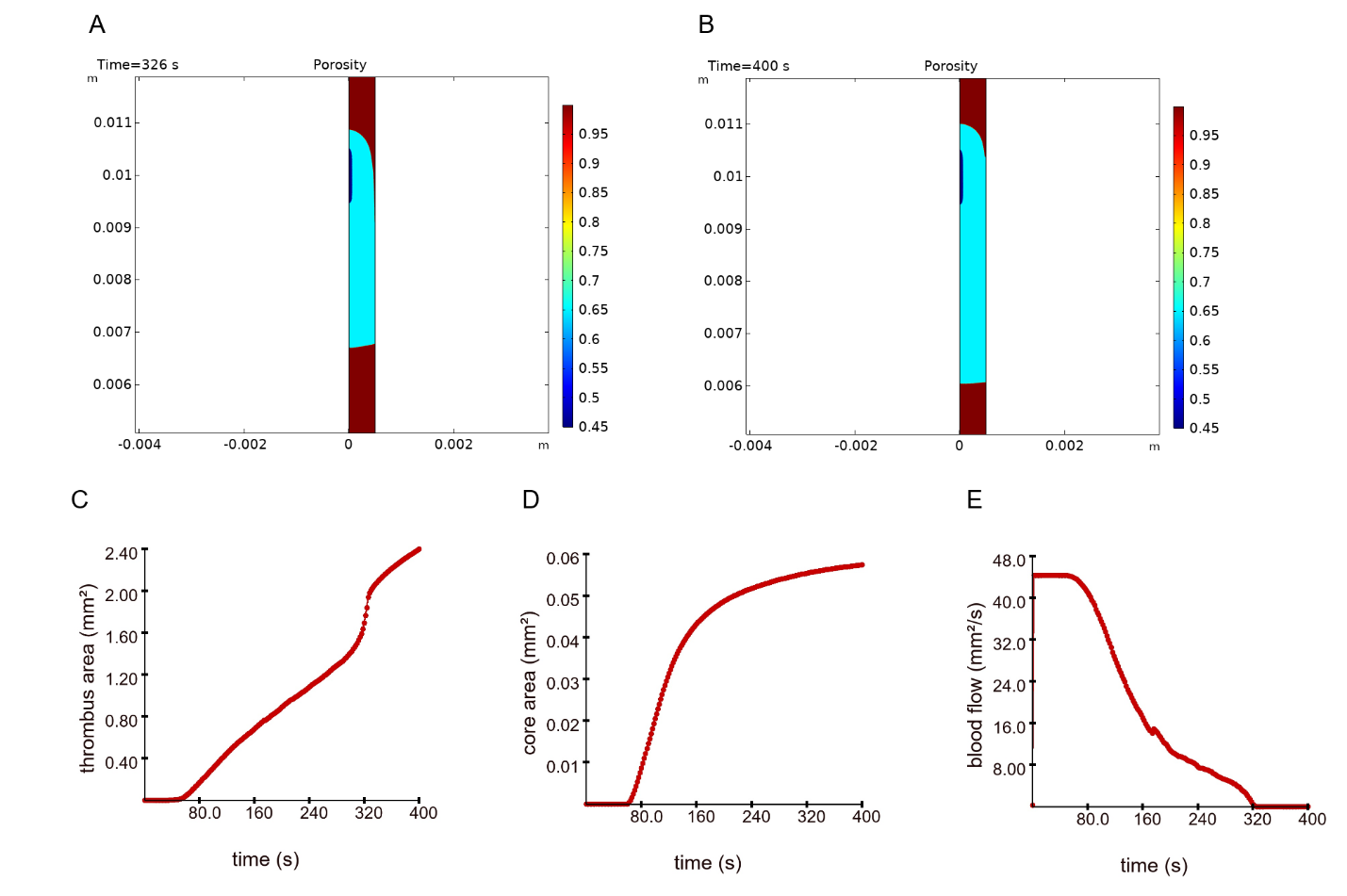

Supplement: S8 Fig — Simulation lasted 400 seconds, thrombin flux was 2 pmol/(m2 ⋅ s). A), B) - Images of thrombus in the model simulation (vessel lumen is brown, shell is blue, core is dark blue). Flow direction was from top to bottom. Color bar shows values of the porosity corresponding to colors on this image. C) Temporal dynamics of the thrombus area in the model simulation. D) Temporal dynamics of thrombus core area in the model simulation. E) Temporal dynamics of blood flow in the model simulation. (TIF) [file pcbi.1014062.s016.tif]

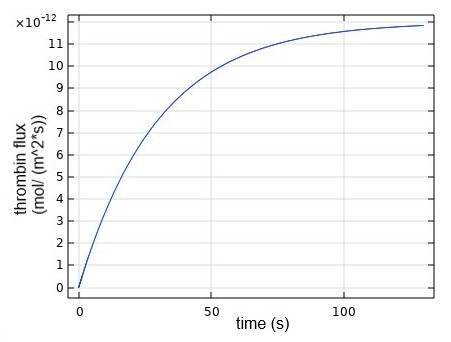

Supplement: S9 Fig — (TIF) [file pcbi.1014062.s017.tif]

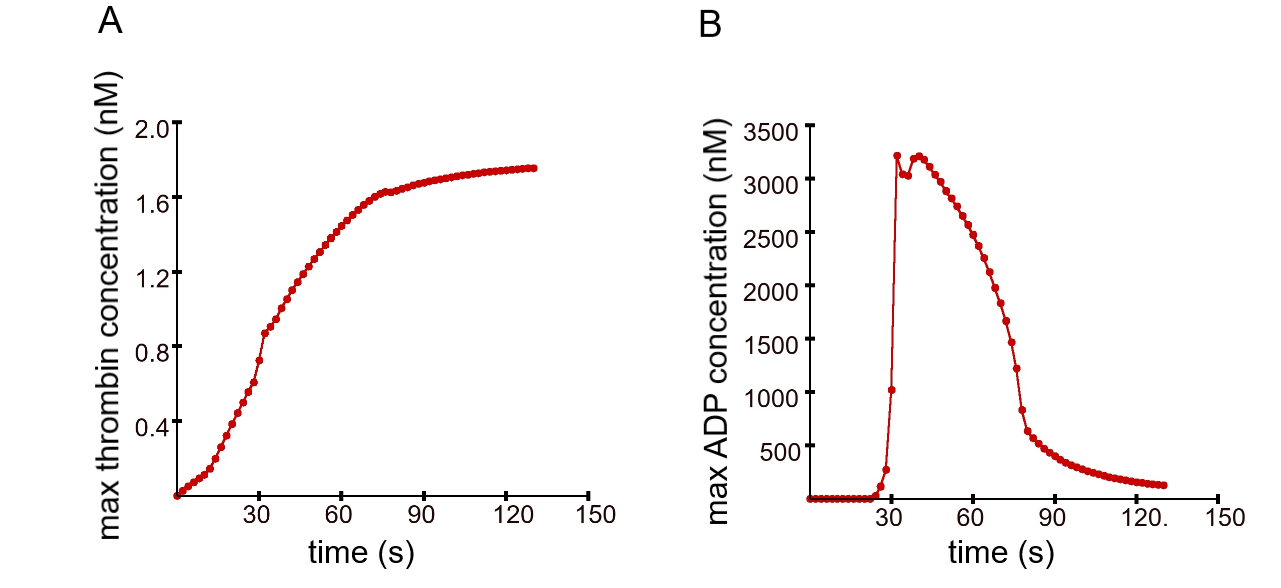

Supplement: S10 Fig — Maximal concentrations of thrombin and ADP inside the whole computational domain (i.e., in thrombus and vessel) were calculated at each time moment. Note significant decrease of ADP concentration by the second minute. (TIF) [file pcbi.1014062.s018.tif]

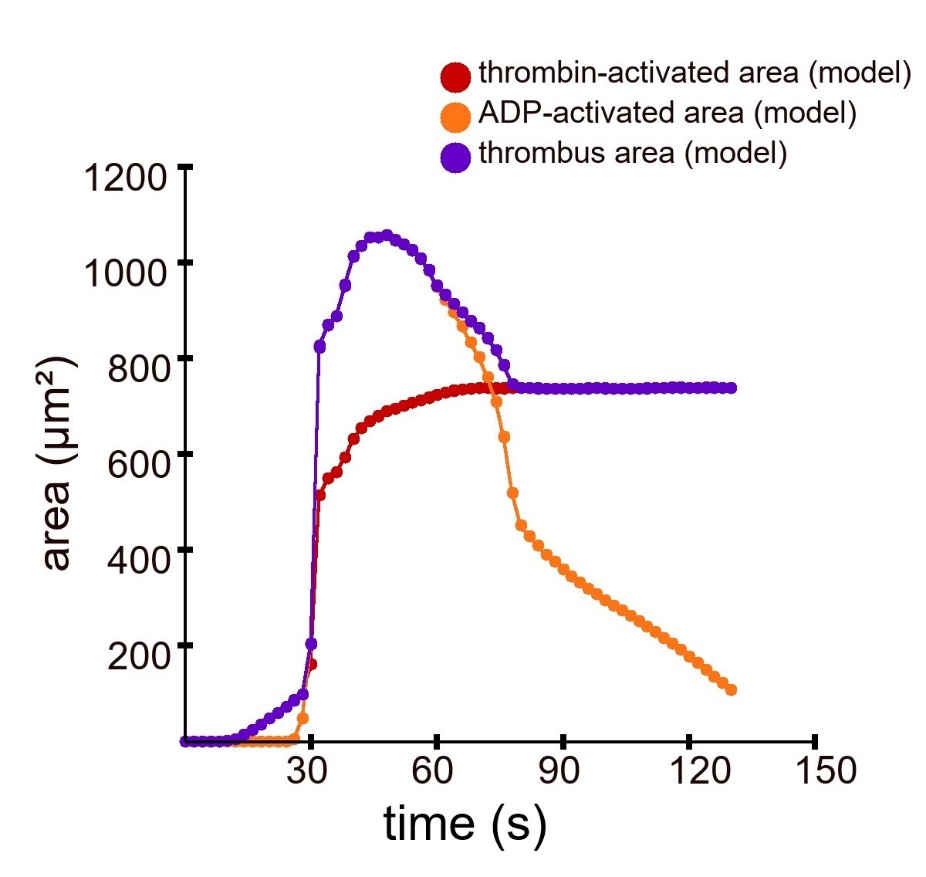

Supplement: S11 Fig — Temporal dynamics of thrombin-activated area (red dots), ADP-activated area (orange dots), and overall thrombus area (blue dots). (TIF) [file pcbi.1014062.s019.tif]

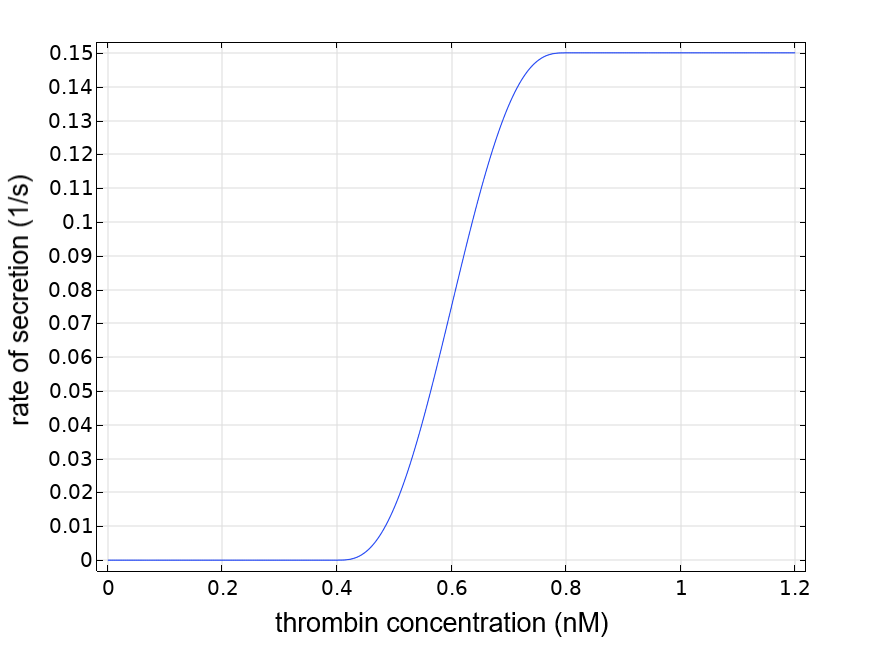

Supplement: S12 Fig — (TIF) [file pcbi.1014062.s020.tif]

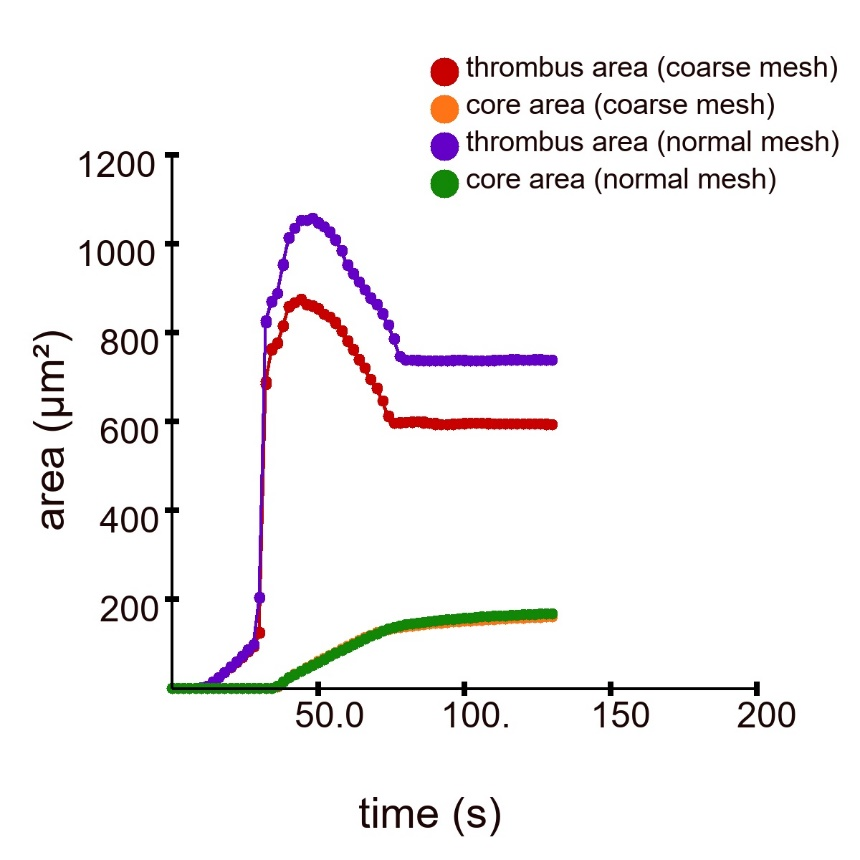

Supplement: S13 Fig — Temporal dynamics of thrombus core area and thrombus area in the model simulation with normal mesh (green and blue dots) and with coarse mesh (orange and red dots). (TIF) [file pcbi.1014062.s021.tif]

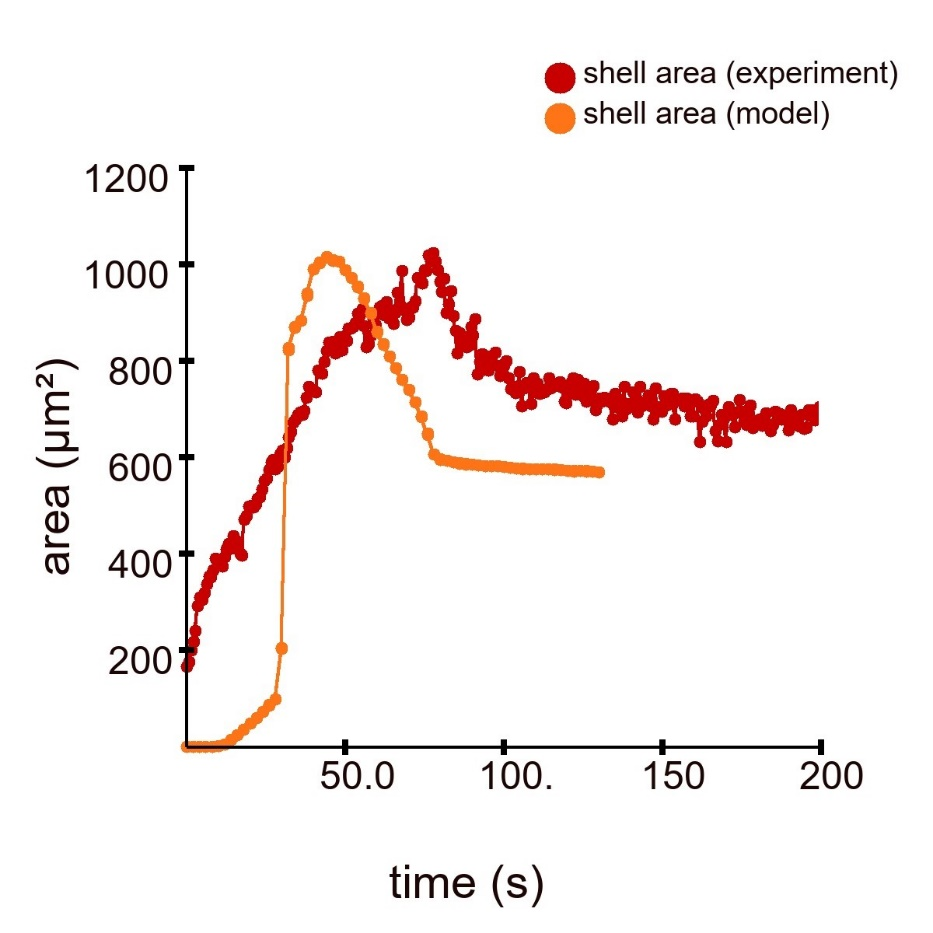

Supplement: S14 Fig — Computations were performed using 3D continuum model in microcirculation. Temporal dynamics of thrombus shell area in the model simulation (orange dots) and in vivo experiment (red dots). Experimental data from Movie A from [11]. Shell area was calculated as the difference between thrombus area and thrombus core area. (TIF) [file pcbi.1014062.s022.tif]

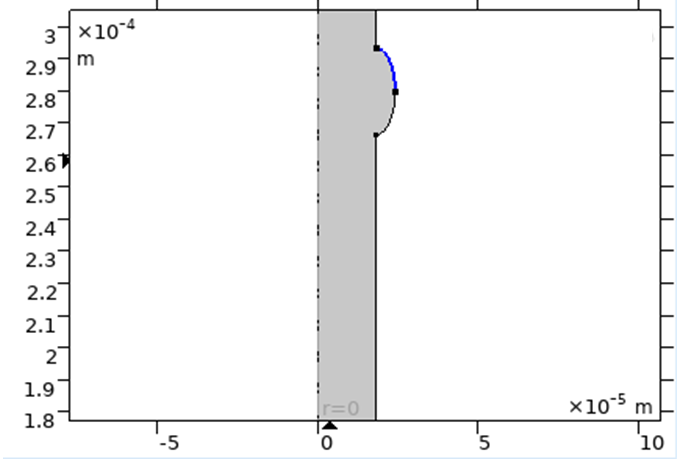

Supplement: S15 Fig — Model geometry. 2D axisymmetric computational domain consisted of a rectangle representing the vessel and an injury site zone. Vessel radius was 18 microns, vessel length was 3060 microns. Injury zone was represented as an ellipse with semi-axes of 13.5 and 6 microns. Center of the ellipse was located on the vessel wall. The zone of thrombin generation is marked in blue. Flow direction was from top to bottom. The dotted line shows the axis of the vessel (symmetry axis). (TIF) [file pcbi.1014062.s023.tif]

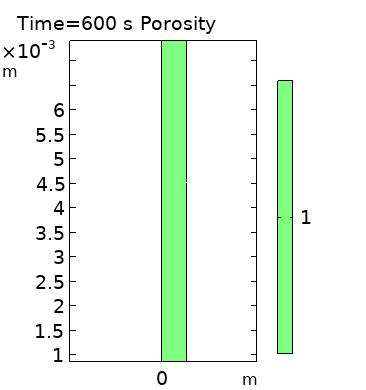

Supplement: S16 Fig — Vessel lumen is green. No thrombus was formed during 600 seconds of the model simulation. Thrombin flux was 1 pmol/(m2 ⋅ s). (TIF) [file pcbi.1014062.s024.tif]
